# Chitosan alchemy: transforming tissue engineering and wound healing

**DOI:** 10.1039/d4ra01594k

**Published:** 2024-06-17

**Authors:** Sheersha Pramanik, Akanksha Aggarwal, Ammar Kadi, Majid Alhomrani, Abdulhakeem S. Alamri, Walaa F. Alsanie, Kanchan Koul, A. Deepak, Stefano Bellucci

**Affiliations:** a Department of Biotechnology, Bhupat and Jyoti Mehta School of Biosciences, Indian Institute of Technology Madras Chennai 600036 Tamil Nadu India; b Department of Biotechnology, Indian Institute of Technology Hyderabad Kandi Sangareddy Telangana 502284 India; c Delhi Institute of Pharmaceutical Sciences and Research, Delhi Pharmaceutical Sciences and Research University New Delhi 110017 India; d Department of Food and Biotechnology, South Ural State University Chelyabinsk 454080 Russia; e Department of Clinical Laboratory Sciences, The Faculty of Applied Medical Sciences, Taif University Taif Saudi Arabia; f Research Centre for Health Sciences, Deanship of Graduate Studies and Scientific Research, Taif University Taif Saudi Arabia; g Department of Physiotherapy, Jain School of Sports Education and Research, Jain University Bangalore Karnataka 560069 India; h Saveetha Institute of Medical and Technical Sciences, Saveetha School of Engineering Chennai Tamil Nadu 600128 India deepakarun@saveetha.com; i 7INFN-Laboratori Nazionali di Frascati Via E. Fermi 54 00044 Frascati Italy stefano.bellucci@lnf.infn.it

## Abstract

Chitosan, a biopolymer acquired from chitin, has emerged as a versatile and favorable material in the domain of tissue engineering and wound healing. Its biocompatibility, biodegradability, and antimicrobial characteristics make it a suitable candidate for these applications. In tissue engineering, chitosan-based formulations have garnered substantial attention as they have the ability to mimic the extracellular matrix, furnishing an optimal microenvironment for cell adhesion, proliferation, and differentiation. In the realm of wound healing, chitosan-based dressings have revealed exceptional characteristics. They maintain a moist wound environment, expedite wound closure, and prevent infections. These formulations provide controlled release mechanisms, assuring sustained delivery of bioactive molecules to the wound area. Chitosan's immunomodulatory properties have also been investigated to govern the inflammatory reaction during wound healing, fostering a balanced healing procedure. In summary, recent progress in chitosan-based formulations portrays a substantial stride in tissue engineering and wound healing. These innovative approaches hold great promise for enhancing patient outcomes, diminishing healing times, and minimizing complications in clinical settings. Continued research and development in this field are anticipated to lead to even more sophisticated chitosan-based formulations for tissue repair and wound management. The integration of chitosan with emergent technologies emphasizes its potential as a cornerstone in the future of regenerative medicine and wound care. Initially, this review provides an outline of sources and unique properties of chitosan, followed by recent signs of progress in chitosan-based formulations for tissue engineering and wound healing, underscoring their potential and innovative strategies.

## Abstract 2

Chitosan, a biopolymer acquired from chitin, has emerged as a versatile and favorable material in the domain of tissue engineering and wound healing.

## Graphical abstract

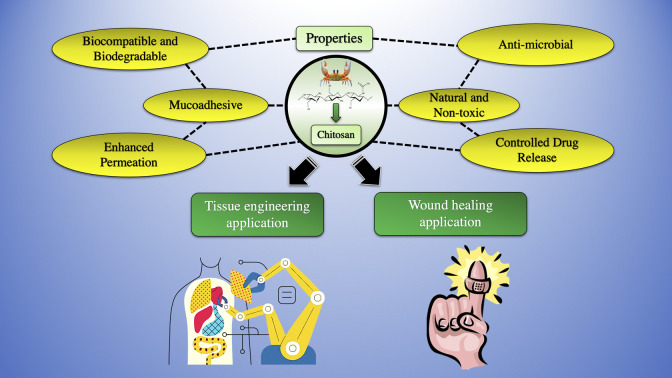

## 1. Introduction

Tissue engineering portrays an advanced field within remedial medicine, initiated from advances in biomaterials research. In essence, this approach comprises the restoration, improvement, and maintenance of damaged tissue or organs by combining cells, biologically active substances, and supporting frameworks. The primary goal of tissue engineering is to develop functional constructs that furnish biological support to injured tissue or organs, assisting their proper healing and regeneration.^1,2^ For example, when treating injuries, various therapeutic strategies have been considered. However, while grafting is an extensively used treatment option, it bears the risk of tissue or organ rejection and transmission of infections. Moreover, it demands additional surgical processes and poses difficulties obtaining healthy donor tissues.^3^

In the present context, tissue engineering carries substantial promise for patients with different injuries. Tissue engineering can be achieved by employing cells (specifically reparative cells such as embryonic stem cells that contribute to the creation of functional tissue), scaffolds (biomaterials that endorse cell's growth and proliferation), or mediators (which encompass bioactive molecules like growth factors or cytokines that guide cells in arranging into appropriately functioning tissue).^4^ Within this regenerative strategy, scaffolds assist as a dual purpose.^5–7^ They behave as temporary three-dimensional frameworks for defining the intended tissues and also function in filling space while regulating signaling molecule's release. To efficiently fulfill these different roles in tissue engineering, scaffolds must demonstrate specific characteristics. These involve compatibility with the encircling tissues to hinder adverse reactions or immune responses, biodegradation at a rate that coordinates with new tissue generation, absence of toxicity and immune-triggering characteristics, mechanical properties that are ideal for the application, and satisfactory porosity and framework for assisting the cells, gases, metabolite, nutrients, and signaling molecules transport both through the scaffold and between the scaffold and the local surroundings.

Several biodegradable polymers have experienced extensive exploration as scaffolds in tissue engineering and wound healing applications. Among them, naturally sourced polymers hold particular importance owing to their biological and chemical similarities to natural tissues, as they are essential elements of living structures.^8,9^ In this connection, chitosan catches the eye as an intriguing candidate with an extensive range of applications and exceptional biological characteristics.^10,11^ These properties encompass biocompatibility, biodegradability into safe by-products, non-toxicity, physiological inactivity, strong affinity towards proteins, fungistatic, anti-tumoral, and anti-cholesteremic characteristics.^12,13^ Not only in tissue engineering, but these unique characteristics, along with wound healing accelerator, antibacterial, and hemostatic properties, make chitosan favorable for wound healing applications.^14^ The assortment of chitosan as a biomaterial is affected, among other factors, by its capability to have its biological, physical, and chemical characteristics regulated and designed in numerous ways, all under mild environments.^15^

## 2. The ambit of the present review

Fundamentally, a review article functions as a comprehensive summary of the present state-of-the-art concerning a definite subject, designed from the accessible research data on that matter. The purpose of this particular review is to shed light on diverse facets of chitosan-based composites. It aims to pinpoint research areas that have observed significant advancements over the past decade in the field of tissue engineering, employing chitosan and related materials as scaffolds. These substances are either utilized independently or in conjunction with other natural or synthetic polymers.

## 3. Structure and properties of chitosan

**Fig. 1:**
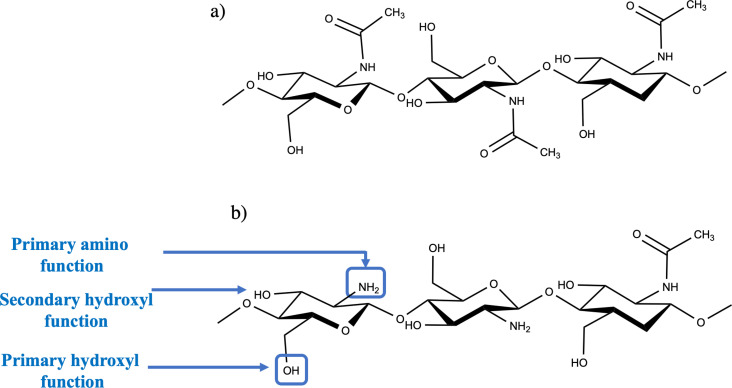
The chemical structure of (a) chitin (b) chitosan.

Chitosan is a linear, semi-crystalline polysaccharide (as displayed in Fig. 1b) made up of units of (1 → 4)-2-acetamido-2-deoxy-β-d-glucan (*N*-acetyl d-glucosamine) and (1 → 4)-2-amino-2-deoxy-β-d-glucan (d-glucosamine).^16^ Chitin is frequently encountered in diverse sources, which include invertebrates such as crustacean shells and insect cuticles. It is also found in certain mushroom coverings, green algae's cell walls, and yeasts.^10,17^ Although chitosan isn't abundantly found in nature, it can be effortlessly acquired *via* the partial deacetylation of a natural polymer referred to as chitin (as shown in Fig. 1b). The degree of deacetylation (DD) of chitosan, which indicates the number of amino groups along its chains, is determined by calculating the ratio of d-glucosamine to the total of d-glucosamine and *N*-acetyl d-glucosamine. To be mentioned as “chitosan,” the deacetylated chitin should include at least 60% d-glucosamine residues^18^ (similar to a 60 deacetylation degree). The deacetylation procedure for chitin can be guided *via* chemical hydrolysis under a strong alkaline environment or enzymatic hydrolysis employing definite enzymes, comprising chitin deacetylase.^19^

Chitosan's characteristics are significantly influenced by the processing conditions it experiences during manufacturing. These conditions play a vital role in regulating DD, which is a key element in deciding its physical, chemical, and biological characteristics. DD is evaluated based on the quantity of unbound amino groups within the polymer chain, and these amino groups impart a positive charge to chitosan. Both the amino and hydroxyl groups assist in its functionality, rendering chitosan a highly reactive polysaccharide. The positive charge inherent in chitosan permits several electrostatic interactions with negatively charged molecules. The processing conditions, along with the extent of functional group generation *via* deacetylation, affect the coupling of these groups, thereby impacting the crystalline nature of chitosan. This, in turn, directly impacts chitosan's solubility in acidic aqueous solutions, an essential property pivotal for its processing.^12,20^ Chitosan stands out as the preferable form of chitin owing to its high solubility in diluted organic acids, making it more available for employment in chemical procedures.^21^ The solubility of chitosan is contingent upon the organization of free amino and *N*-acetyl groups within its framework. When exposed to diluted acid solutions with a pH equal to or less than 6, the free amino groups become protonated, ensuing in a polycationic behaviour for the molecule, and consequently rendering it soluble.^22^ Research has also revealed that when the DD is higher, generally falling within the range of 84% to 90%, the degradation procedure has a tendency to be delayed. Chitosan, with a high DD, exceeding 85%, displays a lower degradation rate when exposed to aqueous conditions and will typically degrade over a longer time, often ranging several months. Contrarily, chitosan with a lower DD, typically varying from 82% to 65%, experiences a faster degradation procedure. Commercially marketed chitosan formulations typically hold DD that falls within 60% to 90%.^23^ It has been reported that chitosan membranes tend to display greater viscosity as their molecular weight is enhanced. This increased viscosity permits for improved control of fluidity within the membranes, which is an essential characteristic for interactions with bio-tissues. Chitosan, owing to its high molecular weight and linear, non-branched framework, assists as an efficient viscosity-enhancing agent in acidic conditions. It operates as a pseudoplastic material, pointing out that its viscosity relies on the degree of agitation or shear force employed on it.^24^ Chitosan scaffolds have the capability to swell when immersed in the liquid medium, and they can hold a definite volume of water absorbed from the surrounding media within their three-dimensional framework. When taking into account their implementation in the biomedical field, it's essential that these scaffolds can absorb fluids from the body, assisting the transport of cells. Moreover, they should permit the appropriate distribution of nutrients, metabolites, and growth factors through the extracellular environment. Chitosan can undergo biodegradation *via* diverse means, which include physical procedures like thermal degradation and chemical processes, such as enzymatic degradation. The rate at which chitosan degrades is inversely associated with its degree of crystallinity, which is impacted by its DD. Therefore, the degradation rate can be modified by controlling the DD in the course of the processing of chitosan.^25^ Chitosan has gained substantial attention as a biomaterial because of its innate origin and diverse beneficial biological characteristics. These properties involve biocompatibility, non-toxicity, non-allergenicity, and biodegradability. Moreover, chitosan displays anti-tumor, antifungal, antioxidant, antibacterial, and anti-inflammatory responses.^26^ In addition, it has been acknowledged for its immunoadjuvant effects, as well as its capability to diminish thrombogenicity and cholesterol levels.^27^ Nevertheless, when employed as a sole material, chitosan shows inadequate mechanical characteristics in moist environments and restricted solubility at pH levels surpassing 7.0. As a result, there has been a quest for diverse strategies to address these drawbacks, either by blending chitosan with other materials or by altering its surface characteristics. Chitosan modification can be achieved *via* diverse physical and chemical methods, which involve complexation,^28^ grafting,^29^ crosslinking,^30^ and blending with polymers,^31^ utilizing functional groups to facilitate the procedure. The antibacterial characteristics of chitosan are typically evaluated utilizing *Escherichia coli* (*E. coli*) as a reference, with the molecular weight and DD of chitosan, as well as the concentration and pH of the medium, performing essential roles.^32^ The antibacterial characteristics tend to reduce from chitosan oligomers to polymers, although it differ depending on the kind of bacteria.^33^ Furthermore, the source of chitosan has been established to impact its antibacterial efficiency. For instance, certain chitosan complexes may combat to penetrate bacterial cells, while others encourage the leakage of intracellular constituents, particularly when chitosan is present at an optimum concentration.^34^ Concerning the degradation of chitosan in the humans, one of the principal mechanisms is enzymatic hydrolytic degradation mediated by lysozymes. This procedure can be efficiently modeled *via in vitro* analysis. The degradation of chitosan by lysozymes includes the cleavage of glycosidic bonds between the polysaccharide units in the polymer. Utilizing this methodology, glucosamine and saccharide are produced as products, which can either be metabolized or stored as proteoglycans in the body.^35^ Chitosan also elicits diverse immunological responses, which involve the promotion of tissue granulation by recruiting fibroblasts, the inhibition of pro-inflammatory cytokines, and type III collagen synthesis. These remarkable characteristics of chitosan suggest its efficacy as an anti-inflammatory, antimicrobial, and wound healing accelerator.^36^ It has been confirmed that chitosan's anti-inflammatory reactions are attributed to the presence of charged moieties in its polymer backbone, which efficiently modulate pro-inflammatory responses. Chitosan has been examined for its promising hemocompatible behavior.^154^ Existing literature indicates that interactions between plasma proteins or blood cells and chitosan's free amino groups could potentially trigger a hemolytic or thrombogenic reaction. Research has suggested that chitosan can be highly thrombogenic, as it has the capability to activate both blood coagulation and complement systems.^37^ Therefore, the exceptional properties and diverse forms of chitosan have paved the way for its comprehensive biomedical applications.

## 4. Advantages of chitosan over other biomaterials

CS is a naturally extracted polysaccharide from crustaceous exoskeletons, showing unique biomaterial properties.^38^ It is well-compatible with the human body and can be excreted easily with non-toxic by-products on enzymatic degradation in the human kidney.^39^ CS possess a cationic nature, thus well interacting with the positively charged membrane of bacterial cell walls, subsequently inhibiting their growth and providing anti-bacterial properties.^40^ CS has been shown to enhance platelet aggregation and is thus suitable as a wound-healing agent. It also aids in sustained drug delivery due to its mucoadhesive properties.^41^ CS also protects the drug against harsh/acidic conditions once inside the body.^42^ The degradability of bio-polymers inside the body depends on the extent of their deacetylation and the availability of attached amino groups.^43^ The charge thus produced can result in toxicity, and through various studies, it has been claimed that the CS and its derivates are not very toxic to human cells. Its versatility remains in its capability to be modified using various functional groups and crosslinking agents, and thus, CS can be customized for desired mechanical properties, degradation rates, and release profiles.^44^ CS also addresses many limitations associated with other polymers, such as polyethylene glycol, which is non-biodegradable and cannot be broken down easily into through natural processes, thus causing long-term safety concerns once inside the body.^45^ Polylactic acid and polyglycolic acid, upon degradation in the body, release toxic by-products such as lactic and glycolic acid, which create an acidic microenvironment causing tissue damage and inflammation.^46^ Polycaprolactone has a slow degradation rate and lacks cell recognition sites, by which CS can stick along in infected areas for a longer time and hinder tissue remodeling and integration.^47^ Polyvinyl alcohol lacks anti-microbial properties and thus demands extra modifications, thus increasing the cost of production.^48^ Gelatin, derived from animal sources, increases the risk of immunogenicity and the risk of transmission of infectious microbes and thus leads to regulatory concerns with its use.^49^ Silicone, after a certain time, needs to be surgically removed because it cannot be naturally degraded and thus brings long-term complications.^46^ Other biotechnological uses of CS can also be summarized for other specific applications. For drug delivery, poly (lactic-*co*-glycolic acid) is highly unstable and occasionally leads to a burst of encapsulated drug and thus counts for ineffective drug delivery potential.^50^ Conversely, CS offers controlled and sustained release with great stability, moisture retention, and hemostatic properties, enhancing patient comfort.^51^ Thus, it can be concluded that CS can provide excellent advantages over other therapeutic bio-materials.

## 5. Chitosan-based formulations for tissue engineering applications

CS, derived from chitin through deacylation, is a marine-based polysaccharide that serves as a structural element in the exoskeletons of crustaceans.^52,53^ It is being explored as a promising biomaterial for TE applications. The CS polymer possesses excellent properties for developing various human tissues, wound healing, and medication delivery. It is biodegradable, effective at adhering to living surfaces, has antibacterial and nonantigenic properties, doesn't elicit an immune reaction, and is thus considered biomimetic. Another significant benefit is the versatility of CS, which may be tailored into gels, nanoparticles, nanofibers, beads, and scaffolds, among other shapes and forms.^54^ They possess significant porosity, and interconnected networks provide adequate rigidity and support for cell growth.^55–57^

### 5.1. CS in bone tissue engineering

Bone, distinguished by its stiffness, hardness, and capacity for regeneration, is an essential part of the body's support system. It functions as a mineral reservoir for microelements such as calcium and phosphate, helps produce blood cells, and, most distinguishingly, provides structural support to the body.^54^ CS biocompatibility is a pivotal factor, as it ensures compatibility with the human body, minimizing the risk of immune reactions or toxicity—a crucial consideration for materials employed in bone tissue engineering (BTE) that must harmoniously interact with living tissues. Additionally, its possession of bioactive properties empowers chitosan to actively induce the growth and differentiation of cells central to bone regeneration, including osteoblasts, thereby facilitating the formation of new bone tissue when utilized in BTE scaffolds. Chitosan's versatility is further highlighted by its ability to be processed into various forms, including sponges, films, and porous scaffolds. This enables the essential diffusion of nutrients and removing waste products—prerequisites for cellular proliferation and tissue rejuvenation. Moreover, chitosan can be tailored to meet the mechanical demands of load-bearing applications in BTE through modification and blending with other materials. Its aptitude as a carrier for bioactive substances, such as growth factors or drugs, adds another dimension to its utility, enabling the gradual release of these agents to foster tissue healing over time.^58^ CS's innate antibacterial properties represent an added advantage, mitigating infection risks at the implantation site—a critical aspect of BTE. Its biodegradable nature ensures gradual metabolization by the body as new tissue forms, minimizing the potential for long-term complications.^59^ The ease with which chitosan can be processed into diverse forms enhances its attractiveness, allowing scaffold customization to align with specific patient requirements and anatomical structures. In summation, CS's multifaceted attributes, including biocompatibility, bioactivity, porosity, mechanical strength, drug delivery capabilities, antibacterial features, and biodegradability, collectively underscore its promise as a pivotal material for advancing bone TE with ongoing research aimed at refining its application in promoting bone regeneration and repair.^60^

**Fig. 2:**
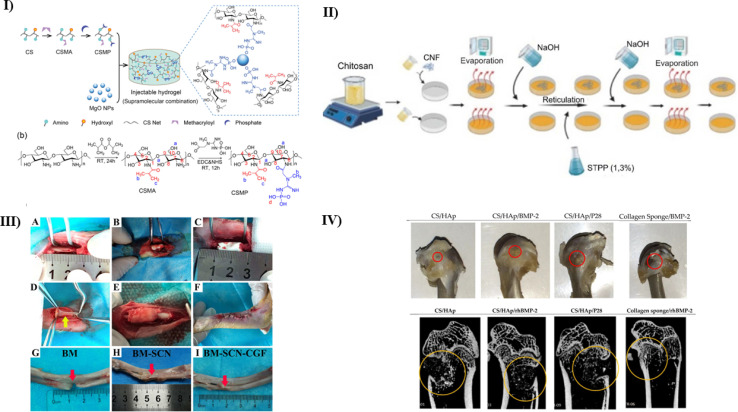
(I) A pictorial representation of (a) fabrication of CSMP-MgO injectable hydrogel, (b) the process of CSMP synthesis reactions, reproduced with permission from ref. 61, copyright 2022, ACS publications; (II) a representation of fabrication of chitosan-CL nanofibers by the method of solvent evaporation using different amounts of CL, reproduced with permission from ref. 62, copyright 2023, IOP publishing; (III) a schematic representation of methodology used for the production of SF/CS/nHAP scaffold to treat critical bone injuries^63^ (IV) (a) *In vivo* analysis depicting defect healing with the use of different types of treatment scaffold used (b) a CT-scan depicting the healing in the mentioned treatment groups after eight weeks of implantation, which represents that CS/HAP/P28 gave better defect closure than other groups used.^64^

Injectable hydrogels derived from natural sources have shown promising results in the repair of critical-sized bone lesions. However, their mechanical, angiogenic, and osteogenic capacities must be improved. Chen *et al.* fabricated CS-based injectable hydrogel containing magnesium oxide nanoparticles (MgO NPs) by adding freshly synthesized water-soluble phosphocreatine-functionalized CS water solution (CSMP) *via* supramolecular combination to overcome these limitations. In a single lyophilization step, methacrylic anhydride and phosphocreatine were grafted onto a CS chain to create water-soluble CS deviate CSMP (as shown in Fig. 2I). This hydrogel's phosphocreatine offers MgO NP sites to mix with to create supramolecular binding. Additionally, it functions as a reservoir for controlling the release of magnesium ions (Mg^2+^). As a result, the pore diameters of the lyophilized CSMP-MgO hydrogels ranged from 50 to 100 μm, and they showed a highly porous scaffold with tiny holes in the pore wall. In contrast to the control groups, where the compression modulus for CSMP was 28.0 kPa and 41.3 kPa for CSMP-MgO (0.5) hydrogels, the CSMP-MgO injectable hydrogels exhibited minimal swelling in DI water (with the lowest swelling ratio reported as 16.0 1.1 g g^−1^). These hydrogels also showed no brittle failure during compression, even at stresses reaching 85% (with a maximum compressive strength of 195.0 kPa). Furthermore, employing a supersaturated calcium phosphate solution, the CSMP-MgO injectable hydrogels stimulated the deposition of calcium phosphate-containing HAP (hydroxyapatite) and tetracalcium phosphate (TTCP) *in vitro*. They did not cause cytotoxicity in MC3T3-E1 cells, and the CSMP-MgO hydrogel promoted osteogenic differentiation in MC3T3-E1 cells, as revealed by the overexpression of osteogenic genes such as BSP, osterix, and OPN. Furthermore, the CSMP-MgO hydrogel has shown outstanding ability in promoting new bone development in calvarial injuries in rats. Therefore, this study concludes that this injectable hydrogel CSMP-MgO has much potential for bone regeneration and should be tested further for clinical trials.^61^

Natural polymeric nano bio-composites show potential in TE to heal injured bone tissues. These substances provide a milieu that resembles ECM (Extracellular matrix), which promotes stem cell development.^65^ In this study, Zanette *et al.* explored a novel cytocompatible nano-bio composite composed of cotton CL (Cellulose) nanofibers and CS polymer to stimulate the differentiation of osteogenic stem cells. Initially, the scaffold was comprehensively characterized, including an analysis of its chemical composition, nano-topography, swelling properties, and mechanical attributes (as depicted in Fig. 2II). The results showed a decrease in elastin modulus as well as mechanical strength with decreased CS matrix content and increased ductility of the scaffold. It was also observed that the CL concentration greatly influenced the swelling and degradation rate inversely. The porosity levels were found to be adequate, indicating solvent retention and absorption. The biological properties of these nanocomposites were assessed to determine their cytocompatibility and potential to induce osteogenic differentiation. For this evaluation, hMSCs (human Mesenchymal Stem Cells) were sourced from exfoliated deciduous teeth. ALP (alkaline phosphatase) result analysis demonstrated that the nanocomposite displayed excellent cytocompatibility and could promote osteogenic differentiation of these cells without requiring chemical inducers and mimicked the biological surface of a bone. This was evident through the increased ALP activity and extracellular matrix mineralization. In conclusion, this study helped to bring a new perspective to synthesizing CS-based biomaterials for bone TE.^62^

In another study, Ray *et al.* fabricated a highly porous and intertwined structure using CS and *Antheraea mylitta* SF using a freeze-drying technique. The protein-to-polysaccharide proportion was kept varied, ranging from a ratio of 9 by 10 to 1 : 1. After careful examination of its mechanical strength, internal structure, and connectivity, the scaffold with a 4 : 2 ratio of SF/CS was found most suitable for bone regeneration. Further to confirm the studies, *in vitro* tests were done using MG-63 cell culture. The artificially created SF80/CH20 scaffold exhibited a more regulated swelling rate of 42.8%, along with substantial adsorption of 0.39 mg of bovine albumin serum per gram during a 24 hours incubation period. Additionally, it showed excellent biocompatibility, cell growth, and F-actin production after a week of cell culturing. *In vivo* histology and fluorochrome studies in rabbit tibia models helped to analyze the good bone regeneration capacity in the scaffold when compared with other control groups. Thus, this study helped to create a scaffold using CS and *Antheraea mylitta* SF for enhanced bone regeneration.^66^

There is a rising emphasis on biomimetic composite scaffolds combined with naturally existing growth factors from the patient's own body to improve the translational success of bone tissue engineering for real-world therapeutic applications. This method is gaining popularity as a contemporary research horizon for treating bone abnormalities. Zhou *et al.* followed vacuum freeze-drying and chemical cross-linking procedures to create three types of SF/CS/nHAP composite biomimetic scaffolds with mass fractions of 3%, 4%, and 5%. *In vitro* studies like ALP staining helped to determine the most hostile nature of the 4% scaffold to provide a good environment for osteogenic differentiation and cell adhesion. *In vivo* rabbit bone defect model treated with the scaffold incorporated with the autologous concentrated growth factor (CGF) helped to identify the synergistic effect of 4% scaffold for bone repair and regeneration (as shown in Fig. 2III). Thus, this study again helped to fabricate another scaffold using CS with another biomaterial in the right proportion for bone tissue regeneration.^63^

A number of studies have been done combining CS and nHAP in different proportions for tissue regeneration. Through extensive research, it is now well known that including a growth factor in the Cs based scaffold provides great biological efficiency. Azaman *et al.* fabricated a CS/HAP scaffold combined with BMP-2 and one of its alike peptides known as P28. The scaffolds were prepared using an off-the-shelf treatment approach following a UV crosslinking procedure. The *in vitro* studies using C2C12 cell culture showed increased ALP activity in scaffolds with BMP-2 and P28 than the negative control. Optical density was also found to be high in both scaffolds, revealing the optimal cell growth and proliferation. The rat femoral condyle defect model was used for *in vivo* studies. A deep examination revealed increased bone density and deposition (as shown in Fig. 2IV). The same was also confirmed with the help of histological studies. Hence, this research presents a novel way to synthesize a scaffold using CS/HAP for bone.^64^

Numerous researchers have investigated various formulations based on CS to promote effective tissue regeneration. For instance, nanomaterials such as CS/SF *Antheraea mylitta*, CS/nano-TiO_2_ sponge,^67^ Nano-TiO_2_ doped CS scaffold,^68^ CS/nHAp/nano-zirconium dioxide,^69^ CS/silicon dioxide/zirconium nanoparticles,^70^ CS/Zirconium oxide,^71^ Human gingival fibroblasts seeded on sphere-shaped nHAp/CS/Gel 3D porous scaffolds,^72^ are some of the recent formulations which have been developed recently.

Although substantial research has been conducted on CS scaffolds reinforced with nanoceramics, unresolved challenges must be tackled before these constructs can progress into clinical trials. Because of their intractable nature in solvents and unique chemical characteristics, toxicity studies on nanoceramics for bone TE applications are crucial. Their high reactivity may cause complications with sample testing since their physical and metabolic expression may change from manufacturing to assessment. Although nanoceramics are novel materials that completely imitate biological systems, their high reactivity and toxicity may significantly impact treatment. As a result, more excellent knowledge of nanoscale surface topography and interactions between nanoceramics and biological systems is paramount at this time.^55,56^

### 5.2. CS in cartilage tissue engineering

CS biocompatibility ensures compatibility with the body's tissues. CS's porous structure facilitates the growth of chondrocytes, essential for cartilage maintenance.^73^ Moreover, it can be employed as a drug delivery system, releasing growth factors to enhance cartilage regeneration. These qualities make CS an excellent choice for constructing scaffolds and implants in TE, holding significant promise for improving treatments for cartilage-related injuries and conditions. Cartilage repair and regeneration pose substantial challenges due to their limited self-healing capacity and associated complications, presenting formidable obstacles in clinical therapy.^74^ Extensive scientific research has confirmed chitosan's well-documented attribute in cartilage tissue engineering—an area of research with profound implications for regenerative medicine. Chitosan's established biocompatibility and capacity to support chondrocyte proliferation render it an ideal substrate for constructing cartilage scaffolds. Empirical evidence has demonstrated that chitosan fosters the production of cartilage-specific extracellular matrix components, including coll and glycosaminoglycans. Additionally, its mechanical properties can be adjusted to mimic the natural stiffness of cartilage. The controlled drug delivery feature of CS facilitates the gradual release of growth factors, thereby further enhancing the process of cartilage regeneration. This scientifically substantiated adaptability positions CS as a critical material in advancing cartilage TE, promising to address joint injuries and degenerative conditions.^75,76^

The use of scaffolding methods and materials that imitate the ECM of host tissue is an essential component of cartilage TE. A study conducted by Ghadirian *et al.* helped to fabricate an electrospun scaffold using CS and polyhydroxybutyrate with the addition of halloysite nanotubes. The physiochemical test to determine the mechanical strength and hydrophilicity of the scaffold revealed good properties of the scaffold containing 3% nanotubes. The FTIR analysis confirmed the formation of new bonds within the scaffold. Additionally, the tensile strength was found to be excellent with decreased mean fiber diameter. The surface contact angle was also found to be decreased with increased surface roughness. Furthermore, it was also observed that the biodegradation was slower in the scaffold. MTT assay revealed significant cell proliferation of chondrocytes after one week of cell culture. Hence this study helped to confirm the fabrication of a new strong CS-based scaffold for cartilage TE.^77^

**Fig. 3:**
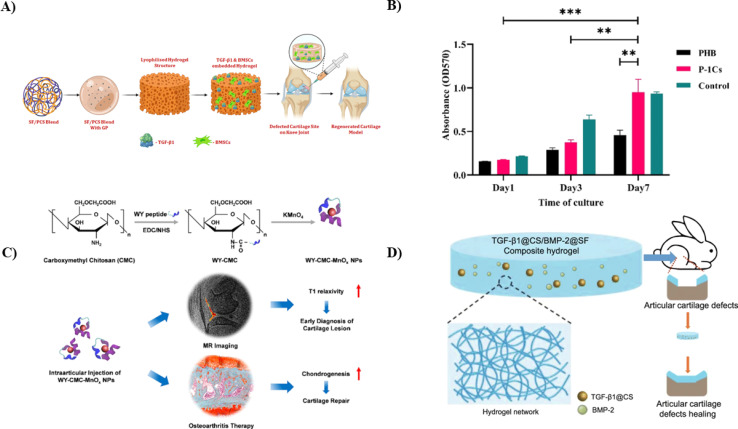
(A) Diagrammatic representation of TGF-β1-encapsulated SF/polylysine-modified injectable hydrogel for cartilage restoration application using BMSCs,^78^ (B) images showing cell proliferation and cell viability of chondrocytes on the nano scaffolds and the control sample on the first, third, and seventh day according to the MTT test, reproduced with permission from ref. 79, copyright 2023, Elsevier; (C) a pictorial representation of the synthesis of WY-CMCS-MnO_*x*_ NPs for cartilage repair as a theragnostic for early-stage osteoarthritis, reproduced with permission from ref. 80, copyright 2022, Elsevier; (D) a schematic illustration to represent the *in vivo* controlled release of TGF-β1 and BMP-2 out of TGF-β1@CS/BMP-2@SF to treat cartilage damage.^81^

Injectable biopolymeric hydrogels, known for their versatility as cell carriers and TE scaffolds, are promising for advancing stem cell-based treatments in cartilage regeneration. Additionally, it's essential for injectable scaffolding biomaterials to exhibit rapid gelation properties while maintaining suitable rheological and mechanical characteristics to support therapeutic applications effectively.^55,56^ Many stem cells, including adipose-derived, bone-derived, and synovium-derived MSCs, have been investigated for cartilage defect repair. It is vital to highlight that immediately implanting cell culture lumps into the defect site might cause problems with cell dispersion, and the underlying structure of these pelleted cells may undergo necrosis at the broken site. The primary aim of the study conducted by Zheng *et al.* was to develop composite hydrogel scaffolds using a combination of CS and Silk Fibroin (SF) materials. These scaffolds were designed to harness their therapeutic potential for cartilage repair through stem cell-based approaches. This was achieved by blending polylysine-modified CS polymer with macromolecular SF, utilizing a thermal-sensitive glycerophosphate. The injectable hydrogel group developed and optimized exhibited excellent biocompatibility with human fibroblast (L929) cells and BMSCs (as illustrated in Fig. 3A). Furthermore, it was observed that the hydrogel could release TGF-β1 sustainably and effectively regulate the expression of genes associated with cartilage-specific processes and inflammation. Finally, the potential for cartilage regeneration was evaluated *in vivo* using Sprague–Dawley rat models through histopathological analysis, revealing promising outcomes for cartilage repair. In summary, it can be confidently stated that the TGF-β1-SF/polylysine-modified CS polymer injectable hydrogel exhibits superior tissue regeneration capabilities both *in vitro* and *in vivo*. This underscores its promising potential as an effective therapy for cartilage regeneration.^78^

Using electrospinning, Amnieh *et al.* synthesized a biomimetic scaffold for cartilage tissue engineering by integrating CS NPs into a polyhydroxybutyrate (PHB) scaffold. The creation of CS NPs between CS in the form of powder and another polymer known as tripolyphosphate (TPP). The PHB scaffolds were assessed for their mechanical properties, hydrophilicity, and fiber diameter while incorporating varying concentrations of CS NPs, ranging from 1% to 5% by weight. The evaluation results demonstrated that the scaffold having one wt% CS NPs exhibited optimal characteristics. This scaffold displayed a significant enhancement in mechanical strength, increasing to 5.2 MPa, and a tensile strength that improved from 5.31% to 12.6%, indicating robust strength. The addition of CS NPs lowered crystallinity and hastened the hydrolytic breakdown process. After seven days of cultivation, the MTT assay analysis indicated a noteworthy increase in chondrocyte proliferation on the scaffold containing one weight of CS NPs compared to pure PHB (as represented in Fig. 3B). These findings suggested that the electrospun CS NPs/PHB scaffold has significant promise for cartilage TE.^79^

Detecting and repairing cartilage lesions early is crucial in osteoarthritis (OA) treatment. However, it is challenging since existing drugs and magnetic resonance (MR) contrast chemicals cannot be detected and repaired simultaneously. To rectify this solution, Lin *et al.* fabricated carboxymethyl CS (CMCS) -assisted manganese oxide nanoparticles (MnO_*x*_ NPs), which were further conjugated with a cartilage targeting peptide known as WYRGRL, also known as WY. The resulting WY-CMCS-MnO_*x*_ NPs (cartilage targeting peptide/carboxymethyl chitosan/manganese oxides nanoparticles) displayed exceptional biological compatibility and optimal T1 relaxivity. Compared with non-cartilage-targeting nanoparticles, the WY-CMCS-MnO_*x*_ NPs significantly improved the quality of MR imaging for cartilage damage due to their tiny size and ability to target cartilage precisely (as shown in Fig. 3C). Conversely, the clinically employed gadolinium-diethylenetriamine pentaacetic acid failed to identify cartilage lesions. Moreover, when tested in rat models *via* intraarticular injection, WY-CMCS-MnO_*x*_ could stimulate chondrogenesis in MSCs, thereby improving OA therapy by facilitating effective cartilage regeneration. These findings underscore the promise of WY-CMCS-MnO_*x*_ NPs for applications in OA.^80^

Cartilage defects are expected near the knee joint, but cartilage possesses limited self-repair capabilities. Hydrogel scaffolds have emerged as up-and-coming tools in tissue engineering to address this issue. Li *et al.* synthesized a developed composite system involving CS nanoparticles combined with transforming growth factor-β1 (TGF-β1) and SF combined with bone morphogenetic protein-2 (BMP-2) to check its ability to repair cartilage knee damage. These systems were thoroughly characterized in size distribution, zeta potential, morphology, and the release profiles of TGF-β1 and BMP-2. To evaluate chondrogenesis, bone marrow stromal cells (BMSCs) were co-cultured with extracts from TGF-β1@CS/BMP-2@SF. Both *in vitro* and *in vivo*, BMSCs displayed normal cell morphology and increased chondrogenic capacity in the presence of TGF-1@CS/BMP-2@SF extracts, as shown by encouraging cell vitality and reducing cartilage abnormalities. Consequently, the hydrogel formulation of TGF-β1@CS/BMP-2@SF, which was developed in this study, effectively enhanced the chondrogenic potential of BMSCs through the controlled release of BMP-2 and TGF-β1 (as illustrated in Fig. 3D). This novel method offers a viable treatment option for articular cartilage abnormalities in knee joints.^81^

Some of the effective formulations used to synthesize different nanocarriers for cartilage tissue engineering include Marine Coll-CS-fucoidan/chondroitin sulfate loaded with primary human cell,^82^ CS/polydroxy butyrate/halloysite nanotubes, CS/PEG silicotugstic acid synthesized using smashed gel combination,^83^ CS/poly(l-glutamic acid) hydrogel,^84^ CS/sodium alginate/bioactive glass,^85^ CMCS/γ-poly-glutamic acid/nanoHXP,^86^ CS/CL NPs/HXP.^87^

### 5.3. CS in dental tissue engineering

CS is gaining recognition in dental TE due to its versatile properties. Its biocompatibility ensures it can be safely used in the oral environment, making it a valuable material for applications like guided tissue and bone regeneration (GTR/GBR). CS-based membranes can create barriers in periodontal defects, promoting the selective growth of specific oral tissues while preventing unwanted cell infiltration. This is particularly beneficial in treating conditions like periodontal disease. CS's antimicrobial attributes are vital in the oral setting, where preventing bacterial colonization is crucial for dental implant success and oral health. It hinders biofilm formation on dental devices, reducing the risk of infections.^88^ Furthermore, CS is an effective drug delivery vehicle for growth factors and antimicrobial agents, allowing controlled release to enhance tissue healing and infection control. Ongoing research in CS-based materials shows promise for improving oral tissue repair and regeneration. By enhancing biocompatibility, antimicrobial properties, and controlled drug delivery, CS could revolutionize dental care, offering more effective and enduring solutions for oral health treatments and benefiting patients and practitioners alike.^89–91^

**Fig. 4:**
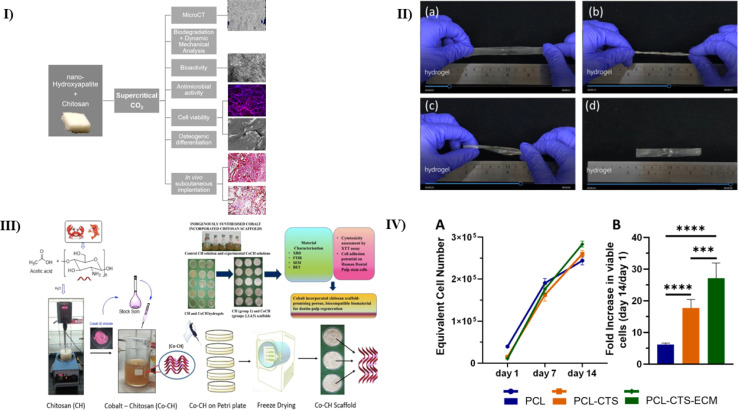
(I) A pictorial representation showing the preparation technique for the CS/nHAP scaffold and the mechanical and *in vitro* analysis used to determine its strength and viability^92^ (II) depiction of thermosensitive hydrogel based on CS and PVA that is flexible and has four different states (a) tensile, (b) torsion, (c) flex, and (d) recovery state,^93^ (III) a diagrammatic representation of the preparation of cobalt-incorporated CS scaffold, reproduced with permission from ref. 94 copyright 2023, Elsevier; (IV) images representing the impacts of electrospun scaffolds on the growth of PDLSCs. day 1 to day 14 PDLSC counts (A) and fold increase (B) in the number of viable cells on PCL, PCL-CTS, and PCL-CTS-ECM electrospun scaffolds at day 14 (compared to day 1).^95^

While bone possesses a natural ability to regenerate, it's worth noting that certain periodontal pathologic and traumatic defects can be of such size that they hinder complete spontaneous regeneration. In this study, Lopes *et al.* manufactured a scaffold using CS and nHAP in the ratio of 3 : 7 using the solvent extraction method using supercritical CO_2_. Micro-CT analysis unveiled a permeable structure with a high degree of interconnection in the nHAP/CS synthesized biomaterial, characterized by a total porosity of 78% and a medium pore size of 200 μm. After 21 days, SEM measurements indicated the development of HAP crystals on the surface of the CS/nHAP scaffold, demonstrating its *in vitro* bioactivity. Furthermore, when put in a PBS solution, incorporating nHAP in the scaffolds resulted in a considerably slower rate of biodegradation relative to a conventional CS scaffold. The viscoelastic characteristics of the scaffolds were validated by dynamic mechanical examination, and the presence of nHAP considerably raised the storage modulus. This implies that the scaffold may aid bone ingrowth in low-load-bearing bone defects. Furthermore, both kinds of scaffolds inhibited *S. aureus* and *E. coli* growth, adhesion, and development of colonies, emphasizing the relevance of CS in the formulation of grafts for usage in the naturally polluted oral environment. In SEM, MG63 cells displayed a normal morphology, adhered well, and proliferated within the porous structure of the biomaterials. This effect was particularly pronounced in the CS/nHAP scaffold, where cells achieved a higher proliferation rate by two weeks. Furthermore, MG63 cells planted within the scaffolds exhibited more osteogenic genes, such as collagen A1, RUNX2, and Sp7, than CS samples. *In vivo* studies in mice with subcutaneous implantation demonstrated that both types of scaffolds exhibited low biodegradability, maintaining their porous structure for up to 5 weeks. Histological analysis revealed a robust and progressive ingrowth of new vessels and collagen, particularly notable in the case of the scaffold, between the 3rd and 5th weeks. In conclusion, this supercritical CO_2_ method has proven effective in producing an affordable and environmentally sustainable CS/nHAP scaffold (as diagrammatically represented in Fig. 4I). This scaffold possesses mechanical, chemical, microstructural, and biocompatibility characteristics that render it a viable alternative for bone grafting in challenging environments, such as those found in periodontitis and peri-implantitis.^92^

The success of dental implants relies on osseointegration, a crucial process. The immune responses, mainly influenced by macrophages upon implantation, play a pivotal role in determining the result of bone healing facilitated by bone-forming cells. One of the challenges in periodontal regeneration surgery is the development of a future periodontal regeneration membrane. This membrane is intended to prevent the invasion of gingiva and fibroblasts into the wound, thus enabling successful regeneration of alveolar bone. Huang *et al.* synthesized a CS/PVA (polyvinyl alcohol) blend thermosensitive hydrogel in this study. The fabricated 2% CS/PVA membranous hydrogel exhibits a notably high swelling ratio, reaching approximately 720% after 60 minutes of incubation. Moreover, it maintains its structural integrity even after a 10 minutes incubation period, making it suitable for surgical sutures. In terms of mechanical properties, the elastic modulus varies for hydrogels with different PVA/CS content: 7.75 ± 1.96, 0.91 ± 0.16, 0.75 ± 0.21, and 0.37 ± 0.06 MPa for 0%, 1%, 2%, and 4% PVA/CS hydrogels, respectively (as shown in Fig. 4II). After undergoing biological decomposition for eight weeks, the remaining weight of the 2% CS/PVA hydrogel was measured at 71.36 ± 0.79%. *In vitro* cytotoxicity assessments revealed that the CS/PVA hydrogel enhanced cell growth and expansion. This method has successfully developed a promising flexible film for use as a periodontal regeneration membrane. So, this research contributes to the conclusion that this manufactured membrane fulfills the function of inhibiting fast fibroblast infiltration into the area of injury and can be used in dental regeneration surgery.^93^

The key to establishing the regeneration of periodontal tissue is to mimic its highly layered and organized structure. The study conducted by Kumar *et al.* helped to fabricate a cobalt-incorporated CS scaffold that can provide optimal cell adhesion and cytotoxicity. The scaffold with varying cobalt concentrations was fabricated and dried. Physiochemical tests using XRD, BET, and SEM-EDX showed excellent porosity and amorphous structure, and FTIR revealed the complex formation of the polymers with the metal. XTT analysis showed the non-cytotoxic nature of the test scaffold. *In vitro* cell-seeding analysis using human dental pulp stem cells showed excellent cell adhesion of scaffold with 1 : 1 100 μmol L^−1^ cobalt chloride with 100 ml 2% CS solution, which highlighted one of the novel findings from this study (as depicted in Fig. 4III). Thus, this study proved to give a biocompatible scaffold for dental tissue engineering with cobalt.^94^

The available scaffold in present in the market for periodontal repair lacks a system to inducible system that could be triggered by physical or chemical stimuli. Santos *et al.* aimed to find a solution and created a nanofibrous scaffold using PCL/CS in combination with lyophilized cell-derived Extracellular matrix (ECM). Equal diameters were observed on SEM examination in both the scaffolds with or without the ECM. Also, the same nitrogen content was observed in the EDX analysis. *In vitro* studies helped to confirm that the incorporation of ECM helped to improve the cell viability and growth of periodontal stem cells by many folds (as represented in Fig. 4IV). Thus, this study helped to introduce a novel method for periodontal tissue repair using lyophilized cell-derived ECM. However, this study further needs to analyze the scaffold activity *in vivo* to fully understand its biocompatibility in the human internal micro-environment.^95^

In another study, Barbosa *et al.* used CS/xanthan gum/HAP to synthesize multi-valent complex membranes in the different mass ratios as 1 : 1 : 0.4, 1 : 1 : 2, and 1 : 1 : 10, respectively. This polysaccharide-based membrane showed complex formation and no change in HAP crystalline structure on FTIR and XRD analysis. TGA showed residual mass higher in scaffold with the highest concentration of HAP. Further analysis showed that an increase in the HAP amount causes a significant increase in surface roughness with more asymmetry. Also, higher *in vitro* proliferation was found in the 1 : 1 : 10 scaffold with the best mechanical and biological property balance. These findings support the possible use of these materials in regeneration procedures and the treatment of periodontal diseases by indicating that adding HA with CS to the membranes can affect the mechanical properties and proliferation for periodontal lesions tissue repair and regrowth.^96^

Some of the recent formulations used for dental and periodontal tissue regeneration bio scaffolds include composites such as carboxymethyl CS/alginate,^97^ CS/HAP/minocycline controlled release system,^98^ CS/oxidized chondroitin sulfate hydrogel,^99^ visible light crosslinked methacrylated carboxymethyl chitosan,^100^ bacterial cellulose, CS, hydroxyapatite, and pomegranate peel extract-based composite scaffold.^101^

### 5.4. CS in neural tissue engineering

**Fig. 5:**
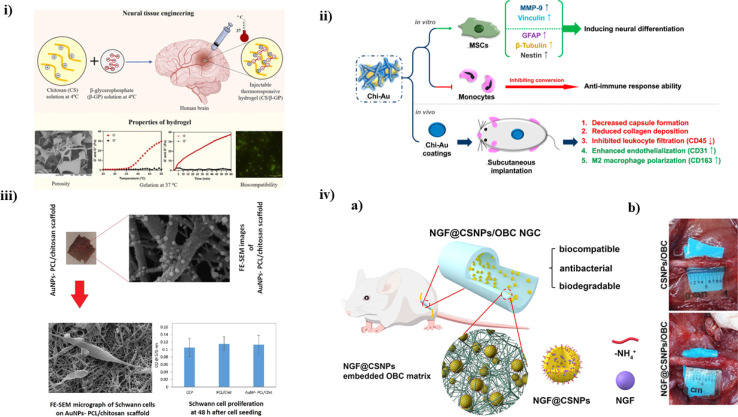
(i) A schematic illustration of fabrication process of CS/β-GP hydrogel and its associated properties, reproduced with permission from ref. 102, copyright 2023, Elsevier; (ii) an overview of CS-Au nanocomposites in both *in vitro* and *in vivo* evaluations for neural tissue engineering with MSCs,^103^ (iii) a pictorial representation summarizing the mechanical and *in vitro* results of AuNPs/PCL/CS-based scaffold to determine its porosity and electroconductivity for nerve TE,^104^ (iv) (a) a diagrammatic representation depicting the composition of NGF/CS NPs embedded matrix, (b) pictures taken during *in vivo* studies concluding that nine weeks of implantation, conduits looked overgrown with freshly produced connective tissue, reproduced with permission from ref. 105, copyright 2021, ACS publications.

Regeneration of brain tissue and recovering lost functions after a CNS injury or disease remains a considerable challenge across the world, with few therapeutic options available. The presence of a blood–brain barrier isolating the nervous system from systemic circulation, as well as the nervous system's limited potential for self-regeneration, are the primary reasons for traditional treatment approaches' failure to restore neural tissue. To come up with a solution, Bhuiyan *et al.* developed a CS/β-glycerophosphate (β-GP)-based hydrogel to check its vitality *in vitro* and determine its mechanical strength. Different ratios of CS and β-GP were mixed together to find a suitable proportion for optimal injectable properties. At physiological temperature and pH, the 0.5%: 3% and 0.75%:3% CS/β-GP hydrogels gelated quickly (3 min and 5 min, respectively), showing their adequate water swelling properties, osmolarity, as well as porosity (as shown in Fig. 5i). *In vitro* tests using PC12 cells helped to establish that the hydrogel had optimal biocompatibility along with the ability for cell growth and proliferation. Thus, this study helped to conclude the usability of this CS-based hydrogel for NTE.^102^

Hydrogels are often used in drug delivery systems (DDS)^106^ and tissue regeneration. In this work, Jafarimanesh *et al.* investigated the potential of valproic acid (Val) enclosed inside a hybrid alginate- CS hydrogel containing CS nanoparticle with/without human endometrial stem cells (hEnSC) for regeneration of spinal cord injury (SCI). The zeta potential, degradation rate, tunability, and swelling ability were studied and evaluated. FTIR analysis helped confirm scaffold formation by detecting peaks at various intensities. It was found that adding Val in CS/Alginate reduced the scaffold size by about 40 nm. 35 Sprague-Dawley rats underwent SCI to assess the synthetic hydrogels' capacity for nerve regeneration. Laminectomy exposed the spinal cords in the T9-T10 region, creating a hemi-section SCI model. The presence of alginate in the hydrogel showed extended degradation time on incubation. Alginate CS hydrogel showed higher swelling capacity than other fabricated hydrogel combinations. The alginate-CS/nCS/hEnSCs/Val may repair injured nerve fibers and histologically limit the vacuolization gaps brought on by SCI. Hence, this study showed that in nerve tissue engineering (NTE) and DDS, the produced alginate-CS/nCS/Val might serve as an excellent polymeric carrier for taurine medicines as a bioactive substrate.^107^

CS is a natural polymer recognized for its potential to promote neural regeneration. In a study by Hung *et al.*, a nanocomposite was developed using CS and varying percentages (25, 50, and 100 ppm) of Au NPs. Upon examination, it was determined that CS mixed with Au NPs (50 ppm) exhibited enhanced biocompatibility *in vitro* experiments. This led to a remarkable decrease in intracellular ROS (Reactive Oxygen Species) production, platelet activation, and monocyte conversion. Moreover, when exposed to CS/Au 50 ppm, MSCs exhibited a higher capacity to form colonies and activate MMPs. The nanocomposites also improved MSCs differentiation, as the lower CD44 expression indicated. Real-time PCR analysis and immunostaining experiments demonstrated that CS-Au 50 ppm substantially elevated the expression of GFAP, β-tubulin, and nestin proteins in MSCs associated with brain development. Furthermore, the enhanced anti-inflammatory and endothelial cell lining formation characteristics of the CS-Au (CS-Au) treatment at a concentration of 50 ppm were evaluated using rats as a model with some subcutaneous implantation. This treatment led to a reduction in collagen synthesis and capsule formation. Notably, there was a noticeable decrease in leukocyte infiltration (CD45) and CD86 expression, indicating a reduction in M1 macrophage polarization. In conclusion, it was demonstrated that adding 50 ppm of Au NPs to a CS polymer improved MSCs' ability to differentiate into neural tissue (as depicted in Fig. 5ii). It has also shown promise as a safe nanomaterial for neural TE.^103^

Peripheral nerve repair continues to pose a substantial clinical challenge, drawing considerable attention. Nerve TE is a cutting-edge therapy strategy that creates an appropriate atmosphere biologically for neuronal cells to solve the obstacles associated with healing. Electrical conductivity and interconnected porosity are two vital characteristics of an efficient scaffold in nerve regeneration. In this study, the primary objective of Pooshidani and colleagues was to fabricate a conductive scaffold characterized by precise control over its porosity. They achieved this by employing PCL and CS. The AuNPs were synthesized *in situ* on the scaffolds using tetrakis (hydroxymethyl), phosphonium chloride (THPC), and formaldehyde. To achieve the desired porosity, varying proportions of polyethylene oxide (PEO) were employed as supportive fibers. Subsequent investigative analysis, conducted using FTIR and field emission-SEM, demonstrated the elimination of PEO from the scaffolds, effectively developing linked porosities. Electrical and elemental tests revealed that gold nanoparticles with an even distribution and an acceptable average diameter were effectively manufactured on the CS/PCL scaffold. The impact of scaffold porosity on their hydrophilic characteristics was shown by contact angle analysis, with porosity levels ranging from 75 to 80 percent, significantly enhancing surface hydrophilicity. Therefore, the outcomes indicated that such conductive scaffolds exhibited no adverse effects and promoted the spindle-shaped morphology of cells with lengthened processes, a characteristic feature of Schwann in cell cultures (as summarized in Fig. 5iii). Consequently, these scaffolds hold promise as potential candidates for applications in nerve tissue engineering.^104^

The most effective treatment for peripheral nerve injury (PNI) is autograft, but subsequent injuries and the lack of donor nerves constrain its use. An appropriate milieu is provided *via* nerve guidance conduits, which might serve as an alternative to autografts by encouraging the regeneration of damaged nerves. To find a solution, Wei *et al.* performed a study where a CS/Nerve growth factor (NGF) solution was introduced under duress to permit a sustained expulsion of NGF, and employing the ion gel approach, NGF-encapsulated CS nanoparticles (nCS) were produced *in situ* in an oxidized bacterial cellulose (OBC) conduit (as depicted in Fig. 5iv(a)). A new nanocomposite comprising CS/NGF//oligomeric calcium phosphate (OBC) was successfully developed. This nanocomposite exhibited antibacterial properties, biodegradability, and a porous microstructure. *In vitro* experiments demonstrated that the nanocomposite enhanced the adherence and escalation of Schwann cells. After four weeks, the ten-millimeter nerve deficit was successfully healed when the nanocomposite was used as NGC to treat rats with sciatic nerve lesions. In the 9th week, the regenerated nerve's histology, diameter, morphology, and functional morphology were equivalent to the autografts, showing that the NGC successfully promoted nerve regeneration and function recovery (as shown in Fig. 5iv(b)). In conclusion, this study concluded that nCS/NGF/OBC nanocomposite is a novel approach to delivering NGF for nerve injuries.^105^

Recently, various studies have been conducted to develop a suitable scaffold for NTE with optimal mechanical strength and physiological viability. Some of the latest examples of the past few years are CS/PEG/multi-walled carbon nanotubes-based composite scaffold (MWCNT) for NTE,^108^ electro-stimulated CS/graphene-based nano-biocomposite for CNS,^109^ ECM modified CS/SF as nerve tissue graft,^110^ biological inert scaffold with chemical altered CS with various functional group such as esterase-activable release for NTE,^111^ nanofibers containing CMCS/modified polyaniline/polyacrylonitrile for MSCs differentiation for neural regeneration,^112^ 3D printed CS/Coll exosomes integrated with human umbilical cord MSCs for neural regeneration,^113^ 3 different scaffold containing CS/hyaluronic acid (HA)/gelatin (GEL) along with CS/Coll and CS/PEG as potential biocomposite for NTE,^114^ CS/aniline/agarose self-gelling hy ogel with drug release factor for NTE.^115^

### 5.5. CS in cardiac tissue engineering

Myocardial infarction (MI) remains a significant societal and healthcare challenge, representing a major cause of global mortality. Cardiovascular illness has remained a prominent cause of mortality, and therapy approaches for myocardial tissue repair have given patients with damaged heart tissue the gift of life and hope. Injectable biocomposites, fabricated from natural and synthetic polymers, capable of locally delivering drugs^116^ or cells to the damaged cardiac tissue for cell differentiation, hold promise in revolutionizing heart-related treatments.

**Fig. 6:**
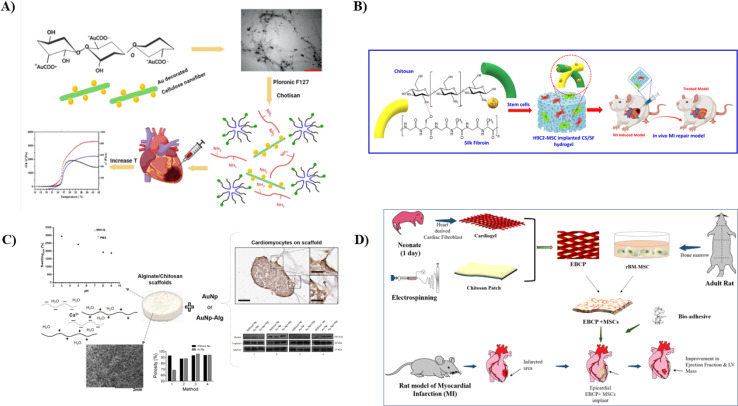
(I) A schematic showing the synthesis of CS/Pluronic/Au nanofibers hydrogel for cardiac tissue repair with increased mechanical strength^117^ (II) a pictorial representation summarizing the preparation of injectable CS/SF NPs hydrogel incorporated with MSCs along with its *in vivo* repair model, reproduced with permission from ref. 118, copyright 2023, Elsevier; (III) a schematic representation of CS/Sodium alginate scaffold with their mechanical and *in vitro* test results with the incorporation of Au NPs,^119^, (IV) a pictorial representation showing the formation of cardiogel-loaded CS patch and its testing *in vivo* adult rat model, reproduced with permission from ref. 120, copyright 2022, Elsevier.

Tohidi *et al.*, therefore, engineered a thermoresponsive and injectable hydrogel for cardiac repair using conjugated CS/poloxamers as a foundation. AuNPs with a typical size distribution of 50 nm were physically attached to oxidized bacterial nanocellulose fibers and mixed at a 1% w/v proportion with a thermosensitive hydrogel (as depicted in Fig. 6A). This adjustment aimed to modify the resulting hydrogel's mechanical characteristics and electrical signal conductance. The formed hydrogels exhibited a porous structure with open pore channels ranging from 50 to 200 micrometers in size. When subjected to increasing temperature with a 5 minutes gelation period, shear rate sweep measurements indicated a reversible transition from a solution to a gel state. The hydrogels displayed an optimal shear modulus ranging and exhibited a shear-thinning behavior. Electrical conductivity assessments revealed that the polymer matrix exhibited significant conductance values in an optimal range. *In vitro* cytocompatibility experiments using H9C2 cells demonstrated high biological compatibility, with cell growth and viability exceeding 90% every 72 hours of incubation. This engineered nanocomposite hydrogel exhibits considerable promise as an injectable bio-compatible material for cardiac TE.^117^

Another study conducted by Shalom *et al.* helped to fabricate a bio composite consisting of CS and Coll. Further, in order to enhance the heart's electrical conductivity, Au NPs were added. Additionally, 5-Azacitidine (5-Aza) was encapsulated to induce cell differentiation. Mechanical studies showed that the synthesized scaffold possessed a hydrodynamically optimal diameter of around 380 nm with optimal electrical conductivity. HPLC and ICP-OES tests validated the loading of 5-Aza and Au NPs in the matrix, whereas FTIR bands corroborated the fingerprint of each composite component. The matrix's biocompatibility was determined using an *in vitro* hemolysis test. *In vivo* animal studies showed the adequate flowability of formulation as an injectable biomaterial. Thus, this study concluded the potential application of this CS/Coll/Au NPs/5-Aza biocomposite for myocardial TE.^121^

Myocardial regeneration by stem cell transplantation and TE has been recognized as a potential strategy for treating myocardial infarction in the past few decades. Wu *et al.* conducted a study to develop a hydrogel containing CS/SF/Au NPs co-cultured with chemically altered MSCs and H9C2 for cardiac tissue repair. The incorporation of NPs was validated using spectral and microscopical analysis. Mechanical strength, drug release factor, and weight loss and drug release factor were found optimal. *In vitro* tests were done using cardiac H9C2 cells and MSCs transplantation with a 2 by 1 ratio of macro-hydrogel with cardiac cells. The gelation time of the macro-hydrogel containing NPs was found to be higher, showing optimal gelling properties with increased intermolecular interactions. No biotoxicity or harmful effects were found during the *in vitro* analysis. *In vivo* study in rat model also helped to determine the restoration of cardiomyocyte fiber and CX43 myocardial indicators (as depicted in Fig. 6B). Hence, this study successfully helped to determine the potential of the abovementioned components to repair myocardial injury and repair myocardial ischemic cells.^118^

In a separate study, Kazemi *et al.* aimed to develop an injectable hydrogel consisting of CS/GEL/glycerol phosphate. It has been hypothesized that the electro-conductive properties of these composites provide an optimal environment for cell growth and proliferation. The SDS emulsion technique was used to make polyaniline/multi-walled carboxylated carbon NTs (PAni/c-MWCNT). This nanocomposite was covered using GEL, and then the hydrogel was coated to avoid their direct interaction. The nanocomposite was uniformly dispersed in the hydrogel to provide uniform electric conductivity. The FTIR studies helped to reveal the interaction peaks to confirm new bond formation. SEM confirmed the uniform distribution of nanostructures. The conductivity of the nanocomposite hydrogel was found optimal. The degradation rate of the fabricated nano-hydrogel was found to be lower, which helped to avoid easy clearance from the body. MTT test using MSCs for 14 days showed biocompatibility, good cell viability, and cell adhesion of the hydrogel. Thus, this conductive thermosensitive injectable hydrogel could be utilized to regenerate heart tissue.^122^

MI, which damages cardiomyocytes, contributes to heart failure (HF) globally. Given the heart's limited natural regenerative abilities, there is a growing interest in the field of cardiac tissue engineering (TE) to create bioactive scaffolds for potential therapeutic applications. Torabi *et al.* thus synthesized a unique injectable and thermosensitive hydrogel loaded with melatonin (active drug) by combining CS, Au NPs, and poly glycerol sebacate (PGS). This hydrogel was developed to advance myocardial tissue engineering. The study involved a thorough examination of the hydrogel. The impact of incorporating GNPs on the electrical conductivity of the hydrogel was also evaluated. Hydrogels were subjected to MTT assays with H9C2 cells to gauge cytotoxicity for up to 7 days. SEM was utilized to assess the shape and structure of seeded cells. The manufacturing conditions for PGS NPs were optimized, resulting in a PGS content of 2.5% w/v and an organic phase to aqueous phase proportion of 1 : 10. The ideal hydrogel displayed a gelation time of 2 minutes. It remained stable for a duration of 20 days. During the initial 12 hours in phosphate-buffered saline, it exhibited a 5% increase in swelling. With uniformly distributed Au NPs, the hydrogel conferred electrical conductivity (1500 μS cm^−1^). Based on the findings from the MTT assay, a concentration of 3.125 μM melatonin was identified as the appropriate level, resulting in a notable improvement in cell viability. These results demonstrated that the hydrogel, created using a combination of CS, Au NPs, and PGS nanoparticles loaded with 3.125 μM melatonin, holds great promise as a scaffold for myocardial TE.^123^

In another study, Beltran-Vargas developed a scaffold to enhance nutrient delivery that could favor more therapeutically active growth for cardiac repair and regrowth. They prepared scaffolds containing different concentrations of sodium alginate and CS, incorporated it with gold nanoparticles, and checked its viability with cardiomyocytes *in vitro*. Using calcium gluconate as a crosslinking agent, the scaffolds (hydrogels) were freeze-dried, and two kinds of metal nanoparticles—gold (AuNp) and gold plus sodium alginate (AuNp + Alg)—were added. Studies on swelling, degradation, permeability, and infrared spectroscopy were used to investigate the scaffolds' physicochemical properties. The resulting scaffolds were extremely porous (>90%) and hydrophilic, with swelling proportions of over 3000% and permeability of approximately 1 × 10^−8^ m^2^ (as shown in Fig. 6C). Furthermore, the suggested scaffolds supported adhesion and spheroid formation, and cardiac markers, including tropomyosin, troponin I, and cardiac myosin were expressed. AuNp + Alg was included, which enhanced the production of cardiac proteins and the proliferation of cells, indicating the potential utility of this 3D CS/sodium alginate/Au NPs hydrogel in cardiac TE.^119^

Cell therapy is one of the most promising methods for heart healing following an injury or infarction. Nevertheless, current methods for delivering MSCs lead to limited donor cell retention and engraftment, reducing therapeutic effectiveness. Here, Sharma *et al.* developed an engineered bio-mimetic cardiogel patch (EBCP) filled with ECM and CS. They have created a new bio-adhesive for suture-free attachment of EBCP to damaged myocardium, inspired by mussels. It uses gelatin catechol and partially oxidized chitosan for wet adhesion through self-crosslinking. *In vitro* experiments with isolated cardiogel showed enhanced cell growth, adhesion, migration, and cardiomyogenic differentiation. EBCP's capacity to shield cells from tissue abrasion in the myocardial infarction (MI) rat model enhances its appeal. Additionally, epicardial implantation of EBCP with MSCs enhances initial cell retention, improves cardiac function recovery, and enhances myocardial tissue restoration. Histological analysis revealed EBCP presence and cell infiltration into the infarcted heart tissue (as illustrated in Fig. 6D). The rapid and straightforward bioadhesive cardio-gel loaded CS patch for myocardial cell renewal, along with significant therapeutic advantages of EBCP, positions it as a promising candidate for heart recovery.^120^

Some recent examples of biopolymer hybrid scaffolds used for cardiac regeneration are CS/polyurethane/GEL/Vicia ervilia/heparin coated nanofibers fabricated using electrospinning for myocardial repair,^124^ CS/PVA/MWNCT nanofibers,^125^ CS/Coll/Tripolyphosphate/Genipin/Glutaraldehyde composite scaffold,^126^ CS/Polypyrrole nanofiber,^127^ CS/gelation hybrid delivering cardiac ECM,^128^ engineered cardiac patch made up pf polypyrrole modified CS for cardiovascular repair,^129^ ternary CS/cardiac ECM/alginate scaffold,^130^ CS/dextran/β-GP for myocardial repair,^131^ electro-conductive scaffold containing CS/graphene oxide.^132^

### 5.6. 3D-printed CS scaffolds in tissue engineering

**Fig. 7:**
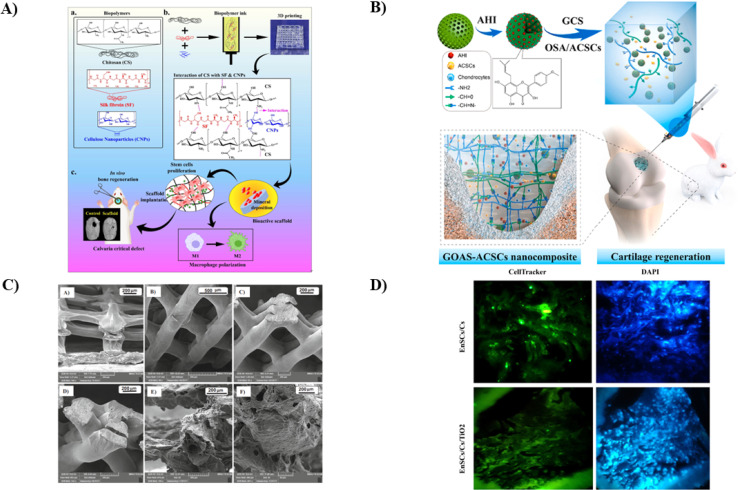
(A) The pictorial representation of CS/SF/CL NPs scaffold (a) the chemical structures of the components used, (b) the steps involved in the fabrication process and (c) the improved application of printed scaffolds for bone regrowth and enhanced cellular activity, reproduced with permission from ref. 133, copyright 2022, Elsevier; (B) a illustration depicting CS hydrogel containing mesoporous SiO_2_ NPs synthesis for cartilage repair and regeneration,^134^ (C) a group of images showed field-emission SEM images taken of 3D CS/Coll/nHA based scaffold taken as different dimensions showing uniform homogenous enhanced porosity and surface characteristics with large pore size, reproduced with permission from ref. 135, copyright 2022, Elsevier; (D) cell tracking images showing that after 8 weeks of transplantation of the CS and Ti oxide containing scaffold where endometrial MSCs were tagged with CMFDA dye to track the transplanted cells' homeostasis in the wounded area, the cells show localization and their contribution in repair in dental tissue, reproduced with permission from ref. 136, copyright 2023, Elsevier.

A biomaterial-based, three-dimensional (3D) scaffold serves as the platform for supporting cell attachment, growth, and differentiation into finished tissue in a tissue engineering construct. The immune microenvironment influenced by biopolymers plays a crucial role in bone repair. Notably, there has yet to be a prior investigation into the osteo-immunomodulatory effects of CS hydrogel scaffolds integrated with cellulose nanoparticles. Patel *et al.* conducted a study to explore how the interactions between cellulose nanoparticles and silk fibroin (SF) affect the immune-modulatory properties of CS biopolymer scaffolds (as shown in Fig. 7A). They employed various spectroscopic techniques to fabricate and assess 3D-printed biodegradable scaffolds comprising cellulose nanoparticles-reinforced CS/silk fibroin (CS/SF/CL NPs). The presence of CL NPs positively impacted the degradation rate of CS/SF/CL NPs. After a 3 days incubation period, it was observed that the scaffold-treated groups exhibited a substantial shift from M1 to M2 macrophage polarization compared to the control, indicating the scaffolds' ability to modulate the immune response. Additionally, they examined the osteo-immunomodulatory effects of these scaffolds on human bone marrow-derived mesenchymal stem cells (hBMSCs) using a conditioned medium produced from macrophages, which showed the migration from M1 to M2 phenotype macrophage. In a rat calvaria defect model, enhanced bone regeneration with high cell density of osteocytes was analyzed using straining studies like H&E, suggesting the potential utility of these manufactured scaffolds in bone-healing applications.^133^

The challenge of repairing articular cartilage remains complicated owing to the intrinsic difficulty of efficiently activating natural healing systems. An increasing focus is being placed on employing implanted stem cells, suitable biomaterials, and bioactive substances in a combined approach to tackle tissue regeneration. This study by Cui *et al.* focused on the rational design of a new injectable nanocomposite, aiming to serve as a sustained release system for the improved cartilage TE. A porous hydrogel made of CS was combined with articular cartilage stem cells (ACSCs) and mesoporous SiO_2_ NPs carrying anhydroicaritin (AHI) to generate the composite material. The synthesized nanocomposite exhibited a significant and prolonged release effect, attributed to the combined influence of the organic hydrogel structure and the mesopore channels within the inorganic mesoporous SiO_2_ NPs. Histological assessments and biomechanical tests revealed that these nanocomposites surpassed in promoting the proliferation and differentiation of ACSCs *in vitro*. Moreover, they demonstrated superior performance in enhancing the synthesis of the ECM *in vivo*. This biocompatible platform efficiently supports cartilage regeneration by continuously releasing AHI, a critical component for promoting the chondrogenesis of ACSCs (as illustrated in Fig. 7B). Consequently, this versatile biocompatible platform substantially enhances cartilage regeneration through the sustained release of AHI. Adding a CS hydrogel containing mesoporous SiO_2_ NPs shows promise as a 3D biomimetic ECM for clinical TE, mainly needed for diagnostic applications.^134^

Crocin (Cro) is a bioactive substance known for its osteoconductive, osteoinductive, and osteogenic differentiation properties, making it a promising choice for developing mechanically reinforced scaffolds in bone TE. This study conducted by Jirofti *et al.* concentrated on a 3D printed matrix that incorporates Cro and is composed of a composite structure comprising CS, Coll (Collagen), and HAP (Hydroxypaptite). The structural characteristics of the scaffolds were assessed through FE-SEM, FTIR, and DSC analyses, which showed the interconnected porous structure of the scaffold (as shown in Fig. 7C). The mechanical properties of the scaffolds were measured by analyzing various forces that significantly affected the scaffold's strength. Incorporating 25 and 50 μl of Cro resulted in a 71% and 74% enhancement in Young's modulus compared to the scaffold without the drug. Furthermore, *in vitro* performance was evaluated by studying degradation rate, swelling ratio, and cell viability, which showed that the scaffold decreases the toxicity of Cro and exhibits biocompatibility. Thus, the findings from this study suggest that the 3D-printed scaffolds loaded with Cro and composed of CS/Coll/HAP exhibit great potential as a promising option for applications in bone TE.^135^

In another study, Hoveizi *et al.* aimed to synthesize a scaffold to define regeneration and direct pulp capping with the help of CS and TiO_2_ NPs by encapsulating human endometrial stem cells (EnSCs) and checking its activity using the Wistar rat model. EnSCs were applied to a 3D CS scaffold incorporating TiO_2_ NPs after collagenase enzyme extraction and purification. Following this, the pulps of the maxillary left number one molars in all rats were exposed, and direct pulp capping was performed using the experimental scaffolds. Glass ionomer was employed as a sealant in all experimental groups and the control group. After eight weeks, the teeth were extracted and subjected to histological evaluation. The CS/EnSCs/TiO_2_ group exhibited a greater quantity and superior quality of dentin than the other groups (as shown in Fig. 7D). The interaction of EnSCs, TiO_2_ NPs, and CS scaffolds resulted in faster and improved dentin production for direct pulp regeneration. In a rat molar tooth model, combining EnSCs and TiO_2_ NPs on a 3D CS scaffold appears to be a viable strategy for dental TE.^136^

One of the crucial elements directly affecting the qualities of the scaffold is the fabrication procedure. Electrospinning is one of several well-established methods for creating nanofibrous scaffolds. Still, it is found that the electrospinning technique to create a three-dimensional (3-D) interconnected macro-pore scaffold is quite challenging. Ehterami *et al.* conducted a study where they developed a highly porous scaffold with the help of CS and poly (l-lactic acid) (PLLA) using liquid–liquid phase separation (LLPS) for nerve TE. Moreover, the impact of various polymeric concentrations on the scaffolds' shape, mechanical characteristics, hydrophilicity, *in vitro* degradation rate, and pH change was assessed. Furthermore, investigating scaffolds as potential options for nerve tissue creation looked at cell adhesion, cell survival, and cell proliferation. In addition to having advantageous structural, porosity, hydrophilicity, mechanical, degradation, and pH alteration characteristics, the CS/PLLA mixture also promoted adhesion, viability, and proliferation of human neuroblastoma cells. It was also found that the different ratios of polymeric concentrations affected both cell responses and their characteristics. So, this study helps conclude that using LLPS, a suitable CS/PLLA 3D nanofibers scaffold was developed, which is promising for nerve tissue engineering.^137^

Some of the other latest research where CS-based composites have been exploited in tissue engineering has been reported in Table 1.

**Table 1:** Recent investigations on CS-based composites for TE

Applications	Nanocarrier type	Materials	*In vitro* cell line	*In vivo* model/area/days	Key features	References
Bone TE	Nanoceramics	CS/graphene oxide/titanium dioxide nanoparticles/blackberry waste extract	—	Three-month-old male wistar rats	After implantation, the animal model exhibited a non-inflammatory response, and there was noticeable bone regrowth, indicating excellent biocompatibility of the implant. At the intraosseous level, an examination revealed the presence of fibrous tissue comprising bundles of type I coll fibers, which appeared to be continuous with the periosteum	138
Bone TE	Nanofibers	nCS/PCL/PVP with veratric acid (VA)	mMSCs	—	The fibers exhibited impressive physiochemical and material characteristics and demonstrated compatibility with mMSCs. Notably, when mMSCs were cultured on these coaxial fibers, VA was pivotal in directing these cells toward osteoblast differentiation, as evidenced by the increased formation of calcium deposits. Additionally, in mMSCs cultured on the PCL/PVP/CS-NP-VA fibers, there was an observed increase in the mRNA expression levels of critical bone-related regulators, including Runx2, as well as other differentiation markers such as ALP, coll type I, as well as osteocalcin	139
Bone TE	Nanocomposite	CS/OctaTMA-POSS NPs	3T3, Saos-2 and MG-63	—	The inclusion of POSS in the material led to changes in surface morphology, primarily by enhancing surface roughness. Moreover, the nanocomposite scaffolds, characterized by a porosity ranging from 82% to 90%, displayed a significant enhancement in the compression modulus, measuring between 78 and 107 kPa, as compared to the control CS group, which registered a value of 56 kPa. These results indicate the compatibility of CS-POSS scaffolds when experimented *in vitro* with different cell lines, including 3T3, Saos-2, and MG-63. Furthermore, the inclusion of POSS exhibited promising effects in enhancing osteoblast proliferation adhesion and elevating osteocalcin secretion, ALP activity, and the biomineralization process within the cells	140
Bone TE	Nanowires	Poly(3-hydroxybutyrate)-CS/alumina nanowires	MG-63 cells	—	Adding 5% alumina increased the fiber diameter, measuring 645.2 ± 192 nm. The bio composite scaffolds that included alumina enhanced their tensile strength tenfold. The scaffolds that included alumina showed superior cell viability	141
Bone TE	Nanofibers	nCS/PCL fibers with sinapic acid (SA)	mMSCs	Rat calvarial bone defect model	The incorporation of 50 μM of SA into nCS within PCL fibers demonstrated a significant enhancement in osteoblast differentiation. SA treatment was found to activate osteogenesis signaling pathways in mMSCs. In a rat calvarial bone defect model with critical-sized defects, including SA within PCL/nCS fibers expedited the process of bone formation. These findings suggest that SA promotes osteoblast differentiation *in vitro* and bone formation *in vivo*. This effect is possibly mediated by activating signaling pathways such as TGF-β1/BMP/Smads/Runx2	142
Nerve TE	Nanofiber hydrogel	CS nanofiber/bioactive peptides (RGI and KLT)	Schwann cells	Rat model of long-distance sciatic nerve defects	The aligned CS nanofibers showed directionally orientation with adequate cell proliferation and secretion of neurotrophic factors by schwann cells. The nanofibers showed increased vascular penetration and nerve regeneration at an early stage of damage. *In vivo* studies showed the promoted nerve regeneration and functional recovery in rats after 12 weeks with improved conduction and motor function recovery	143
Cartilage TE	Bioactive scaffold	CS/silica NPs loaded with kartogenin and platelet-derived growth factor BB	Rabbit BMSCs	24 adult New Zealand white rabbits/femoral trochlear punch/1–3 months	The results show that the scaffold is biocompatible, had a uniform porous structure, excellent water swelling index, promoted cell motility, increased chondrogenic differentiation of BMSCs, could release the growth factor in a sustained manner *in vitro*, and promote cartilage regeneration *in vivo*	144
Tissue engineering	Nano composite hydrogel	CS/aldehyde modified nanocrystalline cellulose/Coll/Au NPs	NIH 3T3 fibroblast cell line	—	Experimental findings revealed that the different molar ratios of Coll/CL and the inclusion of CS-Au content influence the microscopic structure, equilibrium swelling, *in vitro* degradation, and mechanical characteristics of the hydrogels. TEM, FTIR, and TGA revealed spherical morphology, formation of new bonds, and scaffold thermal stability, respectively. Blending these components helped enhance mechanical properties many folds and drastically diminish the degradation rate. An *in vitro* proliferation study for 7 days revealed good cell viability and differentiation abilities. Cytotoxicity analysis demonstrated the efficacy and non-harmful nature of the developed hydrogels	145
Cardiac TE	Nano-dots	CS/Coll/ultra-small graphene quantum dots loaded with hMSCs	hMSCs	Ten male fischer rats	An in-depth physicochemical analysis of the graphene quantum dots and hydrogel combinations confirmed their suitability for applications in cardiac regeneration. *In vitro* assessments of hydrogels combined with hMSCs revealed elevated cell survival rates, increased expression of pro-inflammatory and pro-angiogenic factors, and early indicators of cardiogenesis. Ejection percent, fibrosis area, vascular density, and infarct size all showed improvement from *in vivo* myocardial exams and electrocardiography data. These findings point to significant advancements in improving cardiac regeneration after myocardial infarction	146
Cardiac TE	Nanotubes	CS/PVA/carbon nanotubes	Rat MSCs	—	When examined through SEM, the nanofiber scaffold undergoes mechanical testing cell adhesion, and viability studies showed that the nanofiber containing 1% of C nanotube possessed excellent properties for differentiation of cardiac cells. The enhanced elastic modulus and optimal electrical conductivity were found to be promising. Moreover, the most suitable scaffold was then used to electrically stimulate the differentiation of rat MSCs into cardiomyocytes in the presence of 5-azacytidine, ascorbic acid, and TGF-β. The real-time quantitative PCR (qPCR) data indicated that the expression levels of the cardiac markers Nkx2.5, β-MHC, and troponin I were significantly increased (more than threefold) compared to the control group	147
Skin TE	Nanofibrous scaffold	CS/PLA/ZnO, Fe_3_O_4_ and Au NPs	L929 mouse fibroblasts cell line and human dermal fibroblasts	—	The results of tests on the hybrid materials' conductivity, sensitivity to biodegradation, and water vapor permeability show that they are suitable for skin tissue scaffolds that aid wound healing. *In vitro* tests showed no cytotoxicity and optimal bioactivity, performed through XTT assay. Electro-stimulation was shown to enhance cell growth and thus found helpful for TE	148

## 6. Chitosan-based formulations for wound healing

Wound healing is a particular biological procedure closely associated with the extensive growth and tissue regeneration phenomenon. It progresses through a sequence of interdependent and coordinated phases, wherein diverse cellular and matrix elements combine to restore the integrity of damaged tissue and substitute lost tissue.^149^ This wound healing procedure is intricate and dynamic, generally divided into four phases: hemostasis, inflammation, proliferation, and remodeling.^150^ Considering the intricate nature of the healing procedure, which comprises multiple factors, and the various range of types of wounds, the choice of a suitable wound dressing is of paramount significance.^151,152^ Efficient wound management hinges on an accurate assessment of both the type of wound and the patient, connected with a deep knowledge of the characteristics of potential dressing materials. An ideal wound dressing should assist in fast healing while resulting in minimal inconvenience to the patient. Especially, it should have the capability to control excessive exudate, improve autolytic debridement, and sustain an ideal level of moisture for the healing procedure. Besides these attributes, an ideal wound dressing should have flexibility, adhesivity, and easy to remove characteristics.^153^ Among various polymers investigated as the starting material for ideal wound dressing, chitosan has been in the limelight for its unique properties.

**Fig. 8:**
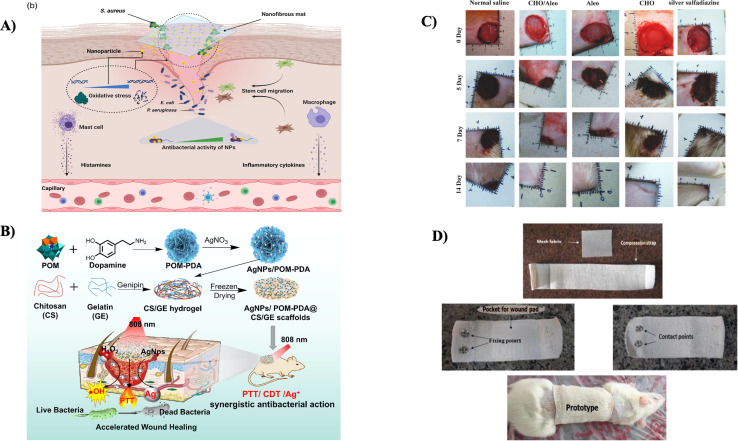
(A) A diagram illustrating nanofibrous composite mats with both antibacterial attributes and the capability to stimulate migration of cells to facilitate wound healing,^154^ (B) a schematic depiction of CS/GE hydrogel scaffolds integrated with hybrid AgNPs/POM-PDA nano-flowers, showcasing their combined effects for faster wound healing *via* photothermal, chemodynamic, and Ag^+^ antibacterial mechanisms, reproduced with permission from ref. 155, copyright 2022, Elsevier; (C) wound healing evaluation: assessing the wound area that remained at day 0, 5, 7, and 14 in diverse groups treated with CHO/Aleo, CHO, Aleo, normal saline (negative control), and silver sulfadiazine (positive control), reproduced with permission from ref. 156, copyright 2022, Elsevier, (D) prototype of wound dressing, reproduced from ref. 157, copyright 2022, Elsevier.

Bacterial wound infections are a prevalent issue within healthcare environments. The excessive employment of antibiotics has contributed to the advent of antibiotic-resistant bacteria. Therefore, there is a pressing necessity for alternative solutions that can both accelerate wound healing and efficiently combat bacterial infections. With this in mind, Bagheri *et al.* developed nanofibers composed of chitosan (CS) and polyethylene oxide (PEO) that were loaded with antibacterial silver and zinc oxide NPs. These nanocomposites had displayed notable antioxidant characteristics and had presented the capability to combat *S. aureus*, *E. coli*, and *P. aeruginosa* infections (the mechanism had been represented in Fig. 8A). Moreover, cell viability evaluation had determined that the ideal concentrations of ZnONPs and AgNPs in the nanofibrous mats were 0.2% w/v and 0.08% w/v, respectively. Significantly, these concentrations did not displayed any cytotoxic effects on fibroblast cells. In addition, the developed scaffold had proven to be compatible with blood, as shown by its effects on coagulation time. Moreover, the researchers had noticed significant migration and proliferation of fibroblast cells at the wound's edge in their wound-healing studies. In conclusion, the developed CS/PEO nanofibrous mats incorporating biocompatible, antioxidant, and antibacterial Ag–ZnO NPs showed favorable outcomes as an efficient wound dressing solution.^154^ This research principally relies on *in vitro* experiments and assays to assess the nanofiber mats properties and efficiency. While these outcomes were beneficial for primary assessment, they may not entirely portray the complicated *in vivo* wound healing conditions. Thus, to determine the practical efficiency of the CS/PEO nano-mats as wound dressings, additional investigations comprising animal models and clinical trials are required.^154^

In a similar context, Zhou *et al.* had created a versatile three-in-one nanocomposite with effective antibacterial characteristics, resembling flower-like structures, by combining silver NPs and phosphotungstic acid-polydopamine nano-flowers (AgNPs/POM-PDA) (as depicted in Fig. 8B). This nanocomposite exhibited photothermal therapy (PTT) capabilities, triggered by near-infrared (NIR) light, which harnessed the photothermal conversion characteristics of polydopamine (PDA). This, in turn, accelerated and regulated the release of silver ions (Ag^+^) from the silver nanoparticles (AgNPs). Moreover, the nanocomposite facilitated chemodynamic therapy (CDT) *via* the catalytic Fenton-like reaction mediated by phosphotungstic acid (POM). The combined treatment approach of PTT, CDT, and Ag^+^ displayed substantial synergistic antibacterial action against both Gram-negative *E. coli* and Gram-positive *S. aureus* bacteria. To develop a multifunctional wound dressing, the researchers embedded the AgNPs/POM-PDA flower-like nanocomposite into a biocompatible hydrogel comprised of chitosan (CS) and gelatin (GE). The synergy between the AgNPs/POM-PDA nanocomposites and the CS/GE hydrogel substantially expedited wound healing *in vivo*, due to the hydrogel's remarkable biocompatibility, water-absorbing characteristics, and breathability. To summarize, the investigation had guided the formation of a versatile agent that efficiently tackled bacterial infections and fostered the wound healing procedure through a multifaceted strategy. But, it is vital to assess the efficiency of the antibacterial treatment against bacteria that have developed resistance to several drugs, as well as to investigate any potential toxicity. This is essential to completely understand the overall therapeutic efficiency and safety.^155^

The procedure of healing diverse types of skin wounds is usually lengthy and frequently includes the risk of bacterial infection and the development of scars. To handle this challenge, Liu *et al.* prepared a biomimetic wound dressing that included electrospun nanofibers loaded with materials that hold dual characteristics of antibacterial action and tissue repair (as shown in Fig. 8B). In this research, they created a composite nanofibrous material comprised of chitosan loaded with Cur@β-CD/AgNPs NPs involving both silver and curcumin. These NPs demonstrated synergistic effects with regard to antibacterial action and their capability to foster wound healing. Their specifically designed silver NPs exhibited efficient antibacterial characteristics against both Gram-negative and Gram-positive bacteria. The CS/Cur@β-CD/AgNPs nanofibers demonstrated a swelling capacity of 432%, signifying their capability to effectively absorb water, which was vital for maintaining a moist environment by efficiently eliminating excess exudates. *In vivo* experiments displayed that the Cur@β-CD/AgNPs chitosan dressing fostered faster rates of wound closure in contrast to the commercially accessible AquacelAg dressing. Moreover, when assessed using Masson's trichrome staining, the developed dressing showed the most uniform distribution of collagen in the wound site. In a nutshell, the Cur@β-CD/AgNPs chitosan nanofibers had the capability to function as an efficient wound dressing with antibacterial properties and the ability to diminish scarring.^158^

In another study, Elshaer *et al.* evaluated the incorporation of clotrimazole (Cz) and fruit extract from Egyptian *Vitis vinifera* into chitosan NPs to improve antifungal efficiency while minimizing adverse reactions. The research displayed that these NPs, referred to as NCs/VJ/Cz, revealed a favorable antifungal activity, resulting in an average diameter of inhibition zone of 74 mm against *C. albicans* and 72 mm against *A. niger*. The developed formulation displayed stability, with a prominent drug EE of 94.7%, a PDI of 0.24, a surface charge of +31, and an average particle diameter of 35.4 nm. The researchers further conducted *ex vivo* and *in vivo* assessments which revealed that NCs/VJ/Cz ointment progressively released the actives in a sustained manner and accomplished complete wound healing and repair of tissue within 7 days of administration. In summary, NCs/VJ/Cz ointment demonstrated assurance as a novel anti-dermatophytic agent with robust wound healing abilities.^159^

In a separate study, the objective of Pourseif *et al.* was to prepare a niosomal platform for the delivery of actives, for example, tetracycline hydrochloride (TCH), to efficiently treat bacterial infections in wounds. In pursuit of this goal, CS was utilized to accomplish the controlled release of drugs while simultaneously offering antibacterial characteristics. The optimization of niosomes encapsulating TCH (TCH-Nio) was conducted employing experimental design methods, emphasizing particle size and EE. Subsequently, the release profile of TCH, as well as the stability of TCH-Nio and TCH-Nio@CS, was assessed. The release rate of TCH from TCH-Nio@CS (72 h) was constantly lower when compared to TCH-Nio under all circumstances. Furthermore, higher temperatures were found to enhance the rate of actives release from these formulations. The size, PDI, and EE of TCH-Nio and TCH-Nio@CS remained more stable at 4 °C in contrast to 25 °C. In terms of antibacterial efficiency, the minimum inhibitory concentration (MIC) values for TCH, TCH-Nio, and TCH-Nio@CS were calculated, with the coated TCH-Nio@CS displaying the most effective antibacterial action against *E. coli*, *P. aeruginosa*, and *S. aureus*. This coating efficiently diminished the initial burst release of TCH from the niosomes (TCH-Nio) and caused in a two-fold increase in antibacterial and anti-biofilm action against the tested bacterial pathogens in contrast to uncoated TCH-Nio, and a four-fold rise compared to the TCH solution. This suggested a synergistic outcome between the encapsulation of niosome and chitosan coating. Eminently, the formulated niosomes revealed no *in vitro* toxicity when tested on human foreskin fibroblast cells (HFF). Both TCH-Nio and TCH-Nio@CS down-regulated the expression of particular biofilm genes in the tested bacteria, for example, csgA, ndvB, and icaA, which partially explained their improved antibacterial activity compared to TCH alone. To summarize, TCH-Nio@CS displayed controlled release of drug and revealed high antibacterial efficiency. But, further *in vivo* studies are essential to determine the true efficacy of the developed formulation for wound healing applications.^160^

Chitosan (CHO)-based hydrogels have the capability to imrpove the wound-healing procedure, and loading Aloe vera into these biopolymeric hydrogels has been proved to further advance wound healing by furnishing protection against a diverse range of microorganisms and improving adhesion and differentiation of cells. Hence, Movaffagh *et al.* developed an enhanced CHO/Aloe hydrogel to foster wound healing in an animal model. The results revealed a significantly higher rate of healing of wounds in the CHO/Aloe group in contrast to other groups at 3, 7, and 14 days (*p* < 0.05). Fig. 8C depicted the top view photographs of the full-thickness wounds appearance of rats that were treated with CHO/Ale, Aleo, and CHO at 0, 5, 7, and 14 days. After 14 days of treatment, the CHO/Aloe gel demonstrated the most efficient wound healing, with excellent tissue tension in contrast to other groups (*p* < 0.05) (as displayed in Fig. 8C). Histological observations showed a prominent difference in the inflammatory reaction between the control and treatment groups after three days of treatment (*p* < 0.05). Moreover, the thickness of the epidermis was substantially more significant in the CHO/Aloe gel group than in the other groups (*p* < 0.05). To summarize, this research had presented an improved topical drug-delivery system utilizing CHO/Aloe hydrogel that had the ability to diminish inflammation over time, thereby fostering faster recovery and enhanced re-epithelialization in the wound-healing process.^156^

As wound dressings have appeared as a promising strategy to improve wound healing procedures, recent attempts have been focused on developing advanced wound dressings utilizing both synthetic and bioactive polymers. In this particular study, Karami *et al.* developed a versatile wound dressing composed of a hydrogel created from carboxymethyl chitosan (CMC) and sodium alginate (Alg), loading a nanostructured lipid carrier (NLC) that encapsulated simvastatin (SIM). The primary objective of this dressing was to assist as a protective barrier against pathogens, fostering the elimination of excess wound exudates, and accelerating the wound healing procedure. Among the diverse dressing composites developed, the hydrogel with a CMC/sodium Alg ratio of 1 : 2 displayed an average pore diameter of roughly 98.44 ± 26.9 μm, with a significant 707 ± 31.9% swelling capacity and a high water vapor transfer rate (WVTR) of 2116 ± 79.2 g m^−2^ per day. These characteristics made it appropriate for absorbing exudates and continuing an optimal level of moisture in the wound environment. The formulated NLC possessed spherical morphology and a uniform particle size distribution of 74.46 ± 7.9 nm. When loaded into the hydrogel, the resulting nanocomposite displayed remarkable antibacterial action against both *E. coli* and *S. aureus*. Furthermore, it revealed no toxicity with L929 mouse fibroblast cells. Crucially, it permitted the controlled and sustained release of the loaded SIM, with the highest rate of release (80%) taking place over a period of 14 days. Overall, the outcomes of this advanced nanocomposite suggested its potential as a favorable candidate for wound dressing, especially for the treatment of diverse chronic skin wounds.^161^ However, the actual performance of the hydrogel *in vivo*, specifically in human subjects or animal models with chronic wounds, is not evaluated. *In vivo* studies are vital to validate the efficiency and safety of the dressing in an actual physiological context.

The advancement of hemostatic products based on chitosan and their implementation in wound healing has long been a pivotal point of investigation in the domain of emergency medicine and biomedicine. In this context, Hasanin *et al.* introduced an advanced design for antimicrobial wound dressings (as depicted in Fig. 8D). These dressings are created from cotton fibers reinforced with stretchable compression straps and safely layered with a tightly woven polyester mesh. Within the cotton layers, the researchers incorporated a biocomposite for wound healing, which consists of chitosan, glycogen, and ZnO NPs (referred to as CG@ZnONPs), and were previously synthesized *via* an environmentally friendly procedure. The size of the ZnO NPs in the CG@ZnONPs composite varied from 30 to 80 nm. The *in vivo* results revealed significantly faster and nearly complete wound healing in the rats treated with the developed dressings, a conclusion further endorsed by histopathological investigation. Furthermore, the dressings displayed substantial antimicrobial action against various pathogenic microorganisms usually found in typical wounds. Thus, these dressings hold promise as a unique biomedical application to foster rapid, successful, and seamless wound healing procedures.^157^ While animal models are beneficial for initial assessments, wound healing procedures and responses in humans may differ substantially. Thus, translating these results to clinical implementations in humans would necessitate additional investigation and validation.

In a separate study, Liu *et al.* investigated the combination of conventional hemostatic chitosan with the prominent inorganic hemostatic material, kaolin, to develop an improved wound dressing. By loading non-toxic, biocompatible, and hydrophilic polyethylene oxide, they fabricated nanofiber membranes referred to as chitosan/polyethylene oxide/kaolin nanofiber membranes (abbreviated as CPKs) *via* electrospinning technology. These membranes demonstrated customizable mechanical characteristics and revealed excellent biocompatibility. In addition, a series of *in vitro* coagulation assays affirmed that CPKs comprising a 10% ratio of kaolin (referred to as CPK10) possessed excellent hemostatic capabilities. Specifically, in the whole blood coagulation time (WBCT) assay, CPK10 showed a substantially shorter hemostatic time (43 ± 1.4 seconds) in contrast to the CPK0 nanofiber membrane (61 ± 2.2 seconds) and QuikClot® Combat Gauze (55.7 ± 1.2 seconds). Following tests on rat liver injuries additionally established that CPK10 efficiently and rapidly ceased bleeding in contrast to other treatments. Furthermore, CPKs were found to enhance the healing of back wounds in rats within 14 days without inducing a substantially inflammatory reaction. Thus, this safe and efficient hemostatic formulation, CPK10, demonstrated itself as a valuable candidate for utilization in pre-hospital medical care.^162^ Though animal models provided initial perceptions, the translation of these outcomes to human applications may include distinct physiological differences that require to be addressed.

The healing of burn wounds can present complex challenges, making it crucial to discover effective methods to enhance the healing process. In this context, Ansari *et al.* aimed to evaluate the healing potential of a hydrogel comprising of antibacterial guar gum (GG), chitosan (CS), and peppermint essential oil (PEO) in treating full-thickness burns in male albino rat models. After introducing full-thickness burns infected *via Staphylococcus* sp. on each rat's dorsum under general anesthesia, diverse wound treatments were employed, involving CS/GG hydrogel, CS gel, CS/PEO hydrogel, and CS/GG/PEO hydrogel. Moreover, the minimum inhibitory concentration of the gel against various *Candida* sp., *E. coli*, and *Staphylococcus* sp. was determined, with values of 9.2, 8.3, 7.6, and 5.7 mg ml^−1^, respectively. Histopathological and histomorphological evaluations, involving contraction of wound, epithelial gap, angiogenesis, thickness of collagen fiber, clustering of fibroblast cells, hyperemia, and inflammatory cell reaction demonstrated that the CS/GG/PEO hydrogel showed the most efficient wound healing performance (roughly 90% wound contraction by the 22nd day). The remarkable performance of the CS/GG/PEO hydrogel in the wound healing process offers valuable understanding for the preparation of clinical antibacterial wound care products.^163^

One of the currently preferred techniques for developing scaffold materials is three-dimensional (3D) bioprinting. This method has achieved popularity owing to its effective and rapid manufacturing procedure.^164^ It includes the deposition of a biomaterial suspension, referred to as “bio-ink,” layer by layer to generate scaffolds with the desirable framework and shape.^165^ The loading of biomaterials, for example, chitosan and their composite polymer blends accomplishes the needs for achieving the required physiological and rheological characteristics, resulting in highly biocompatible and printable bio-inks appropriate for tissue engineering. In this context, Cakmak *et al.* investigated the wound healing characteristics of *Styrax liquidus* (ST), acquired from the *Liquidambar orientalis* (Mill tree), when incorporated into 3D printed scaffolds comprised of polylactic acid (PLA) and chitosan (CS). The n-vitro results demonstrated that the 1% and 2% (by weight) ST-incorporated PLA/CS/ST 3D printed scaffolds displayed an enhancement in cell proliferation. To further examine the impact of ST-incorporated 3D printed scaffolds on the behavior of cells, they performed annexin V/PI double staining, and the results aligned with the *in vitro* assay findings, suggesting low levels of apoptosis induction. Moreover, the wound healing assay revealed that the highest wound closure, in contrast to the control group, was determined in cells treated with PLA/CS/1% ST after 72 h. Thus, this study highlighted the favourability of novel biocompatible ST-loaded 3D printed scaffolds with antimicrobial characteristics for implementation in wound healing.^166^ However, to verify the efficiency and safety of the developed ST-incorporated scaffolds for wound healing, additional *in vivo* experiments comprising animal models would be essential.

Infection and the absence of angiogenesis are the primary elements that impede the healing procedure in chronic wounds. To combat these hurdles, substantial attention has been committed to the advancement of bioactive wound dressings with various functions. One such novelty is a 3D-printed scaffold comprised of carboxymethyl chitosan incorporated with Tacrolimus (TAC-CMC), which has been investigated as a potential bioactive dressing for the healing of chronic wounds. Al-Hashmi *et al.* reported that both CMC and the TAC-CMC scaffolds were found to be biocompatible. Nevertheless, only the TAC-CMC scaffold guided an enhancement in the secretion of vascular endothelial growth factor (VEGF) from fibroblasts. In addition, when subjected to a disk diffusion test, the developed scaffolds demonstrated substantial antibacterial activity against both Gram-negative (*E. coli*) and Gram-positive (*S. aureus*) bacteria. *In vivo* evaluation of the developed wound dressings, both the untreated and bioactive ones, displayed that seven days after the wounds were inflicted, the wounds treated with TAC-CMC had a closure rate of 90.4 ± 2.4%, substantially faster than those treated with the positive control (Comfeel plus®). Histopathological assessments of the treated and untreated wounds established the efficiency of the TAC-CMC scaffold in improving angiogenesis, promoting epidermal regeneration, stimulating fibroblast proliferation, and modulating inflammatory reactions.^167^

*Cordia myxa* fruit extract loaded polycaprolactone/chitosan nanofibers,^168^ Ursolic acid incorporated poly vinyl alcohol/chitosan nanofibers,^169^ Chitosan nanogel treated with probiotic lysate,^170^*Passiflora edulis* leave extract-loaded chitosan hydrogel,^171^ Vitexin incorporated chitosan hydrogel,^172^ chitosan stabilized silver NPs-loaded in chitosan film,^173^ Allantoin and Lidocaine HCL encapsulated chitosan/collagen scaffolds,^174^ Silver sulfadiazine-loaded 3D printed chitosan/alginate hydrogels,^175^ A comparative analysis between chitosan/genipin and chitosan/collagen 3D printed film dressings both loaded with epidermal growth factor,^176^ Pluronic F-127 grafted chitosan/carboxymethyl cellulose hydrogels loaded with nano-curcumin^177^ are some of the recent composites which showed favorable results in wound healing. More recent investigations have been presented in Table 2.

**Table 2:** Current studies on CS-based composites for wound healing

Composite	Preparation method	*In vitro* cell line	*In vivo* model/area	Key features	References
CS/POSS/PEG	Schiff base reaction	HaCAT cells	Ten 8 weeks-old c57/bl mice	The synthesized hydrogel revealed a uniform porous arrangement with optical-mechanical properties, injectability, biocompatibility, and self-renewal abilities. The bio composite also showed cell viability and growth with anti-bacterial properties. *In vivo* studies revealed great self-healing capabilities, excellent endothelial regeneration, and collagen accumulation to promote blood vessel formation by upregulating VEGF overexpression. Thus, this hydrogel is found suitable for diabetic wound healing studies	178
CS-based metal–organic polyhedrons/enzyme hybrid	Crosslinking	L929 cell	Male kunming mice (30–34 g, seven weeks old)/posterior dorsal area	Crosslinking was confirmed using various spectroscopic techniques such as XPS, EDX, FNTIR, and TGA, and it showed the uniform porous interconnected distribution of glucose oxidase and vanadium metal polyhedrons. *In vitro* antibacterial test revealed broad spectrum bactericidal effect of hybrid hydrogel due to the release of free hydroxyl radicals. *In vivo* analysis also showed good healing properties of the hydrogel for its clinical application	179
CS/pectin composite biofilms/Au NPs	Solvent casting method	—	Mice (6–8 weeks old, 30–40 g per mouse)/posterior dorsal area	Physical characterization, mechanical test, and *in vitro* antimicrobial studies of synthesized biofilm showed that adding Au NPs influenced the optical properties of the same along with apparent color. Biofilms showed higher water retention and vapor permeability abilities but low water swelling abilities, low mechanical properties, and decreased water solubility index. *In vivo* experiments helped to conclude the anti-microbial, anti-bacterial, and wound closure potential in 15 days	180
Methacrylate anhydride grafted quaternary ammonium CS/PVA/dopamine	UV-triggered polymerization	L929 cells	Female KM mice/posterior dorsal area	The hydrogel was bacteriostatic against *E. coli* and *S. aureus*, with a ratio of around 90%. Furthermore, dopamine oxidation effectively extracted free radicals and presented the hydrogel with anti-inflammatory, antioxidant properties. It showed that it exhibits properties like ECM and thus showed great potential as a wound-healing dressing	181
CS/poly[2-(methacryloyloxy)ethyl] trimethyl ammonium chloride	—	Human gingival fibroblasts, RAW264.7 macrophage	7 weeks-old Wistar rat/skin excisional defect model	This study aimed to check the immunomodulatory properties of CS for wound healing. *In vitro* studies showed excellent biological compatibility and macrophage migration towards M2 phenotype with optimal antibacterial capabilities. *In vivo* tests helped confirm the enhanced coll deposition and activation of blood vessel formation around the injury, the macrophage polarization towards M2, and the decreased bacterial burden. The study also shows the rebalancing of T helper 17 cells in favor of anti-inflammatory regulatory T cells	182
CMCS/copper NPs/polyphenols	Crosslinking	—	Skin defect model of mice	The results showed that the Schiff base crosslinking reaction provided strong inter-binding, degradable, and self-healing properties. Polyphenols added provided excellent anti-oxidant and anti-bacterial action. The adhesive capabilities to provide good hemostatic adhesivity were also found optimal. *In vivo* studies helped to confirm the tissue formation, polarization of M2 macrophage, and cytokine secretion	183
CS/SF/tea tree oil layer film with sodium ascorbate (SA)-PLGA microspheres	Layer-by-layer self-assembly	NIH3T3 cell	Rat full-thickness skin wound model	PLGA microsphere entrapped in SA produced an anti-oxidant response. The distinctive asymmetric structure of the film provides functional flexibility. MIC studies *in vitro* showed optimal bacteriostatic properties. The biofilm has also been shown to accelerate free radical scavenging. Overall, the film showed suitable adhesion properties with anti-bacterial properties that are optimum for soft tissue regeneration	184
Carboxyethyl CS/oxidized dextran/coll/epidermal growth factor	Schiff base reaction	Mouse embryonic fibroblast (NIH 3T3) cells	—	The introduction of only coll enhanced the mechanical strength of hydrogels by involving Coll's functional –NH_2_ group in the cross-linking process. Additionally, the swelling ratio reached a remarkable 750% for the 3% hydrogel. The introduction of growth factor and coll led to enhanced cell growth and proliferation, with an ability to close the wound in 2 weeks. Staining studies helped to confirm the new tissue growth with collagen distribution	185
CS/ZnO/selenium NPs	Freeze-drying method	Fibroblast NIH 3T3 and HaCaT cell lines	Male albino wistar rats/cut at the dorsal surface	FTIR, XRD, and SEM were used to analyze the chemical properties of the scaffold. They respectively showed new peak formation, the semi-crystalline nature of the lattice, and the semi-crystalline nature of the smooth surface. *In vitro* studies showed that the addition of NPs provided broad spectrum anti-microbial and anti-oxidant properties, with enhanced coll formation, cytocompatibility, and renewal of epithelial tissue with good wound closure potential. *In vivo* studies clearly indicated favorable tissue regeneration abilities within 16 days	186

## 7. Limitations and challenges in chitosan-based formulations research

CS-based biomaterials provide significant advantages over other polymers. However, they possess great limitations and challenges for various biomedical applications. The reliability of the source of biopolymer and its purification method still possesses some contamination concerns for its biomedical use, thus posing challenges in current and future research. Moreover, CS exhibits a variable release profile and occasional burst release of the drug, which raises concerns about the safety of the drug delivery.^187^ Its mucoadhesive property may cause retention of the bio-polymer in the gastrointestinal tract and thus influence the bioavailability of the therapeutic drug for targeted effect.^188^ The synthesis factor, considering its pH, polymer concentration, and ionic strength, affects the ionic equilibrium of the nanoparticles.^189,190^ A clear relationship between the molecular mechanisms behind the size of particles and their nano-biological interactions is still a central axis of research.^191^

Thus, future investigations should include optimization studies for different modifications of CS properties and thorough studies on the proper use of stabilizing agents and conjugation with targeting ligands for controlled release, surface modifications for targeted delivery, and increased bioavailability for better-optimized formulations.^192^ The mechanical strength and structural integrity should also be evaluated well. The CS-based materials also show decreased reproducibility in terms of shape and size.^193^ Thus, it possesses scalability restrictions for commercializing products on an industrial scale with a lot of quality control (QC) checks and regulatory setbacks. The study of the formation of protein corona around the bio-polymer could also be an intensive field of research that will help to overcome the deleterious effect due to undesired binding for drug-delivery systems.^194^ Thus, future investigations for new innovative research with enhanced optimization strategies can help harness the full potential of CS-based formulations.

## 8. Clinical trials

Clinical trials are planned clinical investigations to determine the safety or efficacy of a new treatment/intervention for mass use. Randomized trials produce an unbiased outcome irrespective of age, sex, population, ethnicity, *etc.*^195^ Clinical trials are divided into different stages depending on the main aim of the trial and the number of people participating.^196^ Recently, phase 0 has been added to the list, which helps to determine how a drug/bio-polymer-based product might behave just after laboratory studies. These are micro-dosing studies in which only a very small amount of treatment is given to check the safety of the drugs for human purposes. The lower amount of the drug would be insufficient to produce any therapeutic effect but would be enough to elicit an immune response, producing side effects. Thus, phase 0 is crucial to determine the safety of the treatment.^197^ Further, phase 1 is dose escalation studies in which 20–80 healthy patients are recruited, and a slightly high dose is given. It helps to assess the safety and tolerability of drug usage.^198^ Phase 2 trials are performed on 100–300 participants to check the efficacy of the drug dosage. It helps to find the right dose of the drug and potential side effects. Dummy drugs, or placebos, are also used in clinical trials to assess the true efficacy of a new treatment by comparing its effects against a control group receiving no active treatment. Phase 3 trials aim to determine the best option to cure/manage a particular type of disease and its associated long-term side effects. Lasting up to 4 years, it involves 300–3000 people to check how the treatment will affect the quality of life of an infected/diseased patient. Now, FDA approval is needed to launch the treatment/drug in the market to needy patients. Risk/benefit ratios are accessed for long-term use.^199^ Usually, most treatments have harmful or unwanted effects at this stage, and thus, they must be withdrawn from the market. Chitosan is a bio-polymer based on biomaterial with broad applications in the pharmaceutical and biotechnological sectors.^200^ The stringent regulations enforced by the United States Food and Drug Administration (USFDA) regarding the utilization of CS stem from a profound commitment to consumer safety and product efficacy. These regulations are rooted in a meticulous examination of the extraction procedure and the microbial purity of CS at diverse stages of purification throughout its product development journey. The USFDA's vigilance in this regard has been prompted by a conscientious response to reported instances of allergic reactions related with products containing CS. It's noteworthy that these reactions are predominantly attributed to protein contaminants rather than the CS polymer itself. By upholding rigorous standards, the USFDA endeavors to ensure that any product containing CS meets the highest benchmarks of purity and safety. This commitment emphasizes a dedication to safeguarding public health and fostering consumer confidence in the pharmaceutical and healthcare industries.^201^ By 2022, more than 100 clinical studies were undergoing CS-based biomaterials/NPs, of which 95% were interventional and less than 5% were observational studies.^202^ Clinical trials are essential to performance for larger populations, and the study should not be restricted to smaller numbered samples to reflect the practical usage of chitosan for various usage. The trials are conducted for dental application, eye care, wound care, and bone regeneration. European Union has majorly passed only eye care-based products in the past few years due to safety and purity concerns.^203^ Each year, a significant number of research studies are published on CS-based products; however, only a few of these products progress to clinical trials. This highlights the uncertainty linked to CS-based products, stemming from unpredictable molecular interactions due to the small size of CS nanoparticles and inconsistencies in manufacturing technology.^194^ Regulatory concerns associated with clinical trials are discussed in the succeeding sections, which will explain the requirements needed before conducting a trial on a CS-based bio-product. Table 3 provides an overview of different types of clinical trials based on CS-based biomaterials conducted in recent years.

**Table 3:** The ongoing and completed clinical trials of CS-based formulations [available online: https://clinicaltrials.gov/]

Identifier	Study title	Recruitment status	Subjects/participants	Composite/drugs	Route of administration/delivery method	Phase	Main features
NCT02081885	Tricalcium phosphate and chitosan as bone regenerator *versus* autologous graft in surgery for mandibular fracture	Completed (actual study completion date: 2017–01)	24	CS/tricalcium phosphate	Inserted during surgery	Phase 3	After a mandibular fracture causing bone loss, challenges arise, categorized into functional (*e.g.*, lower lip incompetence, difficulty chewing) and cosmetic (*e.g.*, facial asymmetry). Research into biomaterials aims to improve outcomes, addressing both functional and aesthetic concerns in mandibular reconstruction
NCT03588351	Chitosan, chitosan nanoparticles, and chlorhexidine gluconate, as intra canal medicaments in primary teeth	Unknown (estimated study completion date: 2018-08-30)	Not provided	CS NPs/chlorhexidine gluconate	Intracanal medicaments	Not applicable	The studies has consistently investigated the effects of chitosan as new alternative when used as intra canal medicaments in necrotic primary molars
NCT05661708	Use of chitosan powder in loop electrosurgical excision procedure (LEEP)	Completed (actual study completion date: 2024-05-17)	130	CS powder	Electrosurgical excision	Phase 4	LEEP can lead to postoperative vaginal bleeding (PVB), affecting recovery and daily life. Chitosan, with its hemostatic and wound-healing properties, is being evaluated in a randomized trial to assess its impact on PVB and wound healing following LEEP
NCT05773911	Treatment of advanced periodontitis with a chitosan brush and a chitosan gel (chitosangel)	Recruiting (estimated study completion date: 2024-12-31)	40	CS gel/brush	Sub gingivally	Not applicable	The study aims to evaluate the effectiveness of a chitosan brush, with or without chitosan gel, as a non-surgical treatment for patients with advanced periodontal disease who have not responded well to conventional treatments. The goal is to prevent further bone loss around the teeth
NCT03280849	Chitosan scaffold for sellar floor repair in endoscopic endonasal transsphenoidal surgery	Completed (actual study completion date: 2017–02)	1	CS bilaminar scaffold	Endonasal transsphenoidal surgery	Not applicable	A 65 year-old right-handed female experienced progressive bilateral temporal visual loss over 10 months. MRI revealed a sellar lesion compressing the optic chiasm. She underwent endoscopic endonasal transsphenoidal surgery, using a novel bilaminar chitosan scaffold for sellar floor closure. After two years, her vision returned to normal, with no evidence of the lesion on postoperative MRI and no complications
NCT04840147	A comparison of JointRep® and microfracture in repair of cartilage lesions on the femoral condyle or trochlea, (JMAC)	Recruiting (estimated study completion date: 2025–12)	185	CS-based hydrogel for cartilage repair	Injectable thermo-gelling aqueous composition	Not applicable	The study aims to assess if JointRep® combined with microfracture outperforms microfracture alone in treating symptomatic focal articular cartilage lesions in the knee, exploring Chitosan's potential for enhancing cartilage repair
NCT05333211	Ortho-R® for rotator cuff repair compared with standard of care rotator cuff repair without Ortho-R®	Recruiting (estimated study completion date: 2024–05)	78	CS/trehalose/calcium chloride	Freeze-dried drug/biologic	Phase 1 and 2	A phase I/II, multi-center, prospective, blinded 2-arm, parallel design, randomized controlled study comparing ortho-R/PRP combination and standard care for rotator cuff repair over 12 months. Three training cases precede randomized enrollment; adverse events monitored. Ortho-R® is a freeze-dried drug/biologic combination product containing a chitosan component and other agents
NCT01246895	Follow-up study evaluating the long term safety and efficacy of BST-cargel and microfracture repair of the knee	Completed with results	67	CS-based medical device	Applied to micro fractured lesion	—	BST-CarGel® has demonstrated efficacy as a mid-term solution for repairing cartilage. After 5 years, it showed consistently better repair tissue in both quantity and quality compared to using microfracture alone. The clinical improvement seen with BST-CarGel® combined with microfracture was notably significant compared to the initial conditions
NCT00314236	Trial comparing BST-CarGel and microfracture in repair of articular cartilage lesions in the knee	Completed with results	80	CS-based gel	Application to micro fractured lesion	Not applicable	After twelve months, BST-CarGel treatment exhibited superior lesion filling and repair tissue quality in comparison to microfracture treatment alone. Both groups showed equivalent clinical benefits at this time point, with similar safety profiles
NCT01895933	Efficacy and safety of the investigational device, SurgiShield anti-adhesion barrier gel	Completed (actual study completion date: 2013–04)	33	CS-based solution	Injected at the infected site	Not applicable	The aim of this clinical trial is to assess the impact, effectiveness, and safety of SurgiShield, an adhesion inhibitor formulated with chitosan, in promoting wound healing following endoscopic sinus surgery
NCT05743283	Efficacy of SynEx wound rinse in civilian surrogates of combat injury wounds	Recruiting (estimated study completion date: 2025–09)	100	CS/arginine/sorbitol powder- SynEx wound cleanser	The saline solution was applied tropically	Not applicable	This interventional study aims to evaluate SynEx wound cleanser against standard care (saline) for traumatic wounds. Participants with gunshot, penetrating, or burn wounds will attend up to four study visits, utilize the designated wound cleanser, and provide feedback through brief surveys regarding their wound healing progress
NCT02668055	Slow-release Tb4 collagen and chitosan porous sponge scaffolds skin substitute treatment is difficult to heal wounds (TB4)	Completed (actual study completion date: 2015–12)	20	CS/collagen	Scaffold	Phase 1	Assessing the efficacy of slow-release Tb4 collagen and chitosan porous sponge scaffolds as skin substitutes through clinical trials for treating challenging wounds, ensuring safety over a 6 months period
NCT04211597	EGF-loaded chitosan to facilitate healing and prevent scar formation of cesarean wound	Completed (actual study completion date: 2018-12-28)	60	CS/microencapsulated recombinant human EGF	Wound healing dressing	Not applicable	This study investigates the effect of microencapsulated recombinant human EGF (Me-EGF) combined with silicone gel on scar prevention post-cesarean section

## 9. Regulatory landscape and challenges associated with translating chitosan-based formulations from research to market

CS mucoadhesive properties, pH-responsive behavior, and anti-tumoral, anti-microbial, and anti-immunogenic abilities make it a suitable candidate for the manufacture of large industrial-scale multifunctional products.^204,205^ Chemical and structural modifications help to optimize the properties of products as desired. The Food and Drug Administration, USA, has recently recognized chitosan within GRAS status (generally recognized as safe) to market therapeutics b. The mechanical strength and structural integrity should also be evaluated well. The CS-based materials also show decreased reproducibility in terms of shape and size ased on this bio-polymer with government approval. Till 2022, a total of 3650 patents were recorded from the cosmetic, biotechnological, and pharmaceutical sectors, and it is estimated that the CS global market will reach up to 7.06 billion by 2030 with an annual growth of 20% from now.^206^ However, the translation of CS-based products into the market is still unsatisfactory and requires an in-depth evaluation of its risk-benefit analysis in different lines of research.^194^ The regulatory agencies play an essential role between academic universities and industries in preparing a well-characterized product from research lab to market. During testing, some cases have reported the influence of size and surface charge on CS nanoparticles affecting biological properties.^207^ The current axis of research is based on their colloidal properties (*e.g.*, hydrodynamic and charge properties) and supramolecular interactions.^208^ The macromixing of different active ingredients and excipients in accurate ratios poses a challenge for designing manufacturing operations with accuracy. Malfunctioning of any kind may lead to unstable products and batches with significant variability.^209^ Regulatory agencies regulate a therapeutic product's safety and desired response while eliminating its harmful and undesired effects. CS, which comes from animal sources, should undergo rigorous microbial tests to remove animal-borne contamination.^210^ The extraction methods should be optimized to eliminate any microbial contaminations.^211^ Regulatory agencies should impose critical quality attributes/parameters to monitor the quality/sterility of the product.^212^ The major concerns and challenges associated with translating the research based on it to market lie can be summarized in 3 categories. First, to know the exact mechanism of biological interactions of CS-based biopolymer within a living human body; second, to learn more about the technological aspects of its production to scale up; and the development of regulatory parameters for its quality control checks.^213^ In summary, we can conclude that quality control assays, specialized toxicology studies, and lack of suitable standards and dedicated regulatory guidelines are some of the main regulatory challenges associated with translating chitosan-based formulations from research to market and need monitoring. Evaluating the characteristics of chitosan is crucial, specifically considering the degree of deacetylation, which substantially influences its chemical behavior and capability to adhere to biological surfaces. Notably, there exists a clear relationship between the degree of deacetylation and the viscosity of chitosan solutions.

So, it can be concluded that while chitosan-based formulations hold significant potential in biotechnological applications. Addressing their limitations and challenges through innovative research, optimization strategies, advanced technologies, and interdisciplinary collaborations will be crucial for harnessing the full therapeutic potential of chitosan and accelerating its clinical translation, commercialization, and impact on global healthcare.

## 10. Conclusion and future directions

Chitosan-based formulations have made extraordinary strides in the domain of tissue engineering and wound healing, holding enormous promise for the future of regenerative medicine. As we infer this investigation of recent progress in chitosan-based formulations, it is evident that these biomaterials have developed into versatile tools with multifaceted applications. Chitosan, a natural biopolymer acquired from chitin, has amassed substantial attention for its innate properties that make it a great candidate for tissue engineering and wound healing. Biocompatibility, biodegradability, antimicrobial action, and immunomodulatory effects are some of the noteworthy attributes that have provided its prominence. In the domain of tissue engineering, chitosan-based formulations have appeared as critical tools for producing a conducive microenvironment for attachment, proliferation, and differentiation of cells. These scaffolds can be tailored to resemble the innate ECM and provide unique benefits in regenerating diverse tissues, such as bone, cartilage, skin, *etc.* Novelties in scaffold design, involving 3D printing technology, have permitted precise control over the structure, improving regeneration of specific tissue. Moreover, chitosan's versatility expands to its role in drug delivery systems within these scaffolds. It assists the controlled release of bioactive, comprising growth factors and therapeutic molecules, to improve tissue regeneration and expedite the healing procedure. In the realm of wound healing, chitosan-based dressings have revealed exceptional abilities, such as maintaining a moist wound environment, preventing infections, and promoting rapid wound closure. Furthermore, the immunomodulatory characteristics of chitosan perform a crucial role in controlling the inflammatory reaction during wound healing, fostering a balanced and effective healing procedure.

Chitosan alone often lacks the mechanical strength imperative for load-bearing tissues, making it inappropriate for applications in areas like BTE. Thus, blending chitosan with other biocompatible and biodegradable polymers, such as collagen, alginate, or synthetic polymers like polycaprolactone (PCL), can improve mechanical strength, enhance biocompatibility, and furnish an extensive range of degradation rates. Incorporating nanoparticles or nanofillers (*e.g.*, HA, graphene) into the chitosan matrix also enhances mechanical characteristics, controls degradation, and advances tissue regeneration. The researchers have also explored diverse chemical modifications of chitosan, for example, grafting with functional groups or crosslinking, to enhance its poor solubility, stability, and control over degradation rates. While chitosan-based biomaterials and blends have found commercial applications in wound dressings, there is still a long way to go in optimizing the characteristics of chitosan blends. The main hurdle in blending chitosan with polymers lies in its insolubility and poor mechanical properties. The mechanisms causing the antibacterial activity and wound healing procedure of chitosan are well-known, but complications arise when dealing with chitosan composites/blends. Understanding the mechanisms behind the healing and antibacterial phenomena remains a principal challenge for forthcoming research on chitosan-based formulations. Furthermore, since the properties of chitosan-based formulations are composition-dependent, the determination of the optimal weight ratio of constituents is an essential factor in engineering such systems. Designing materials for wound dressing with optimum absorption of exudate and hindrance of microbial growth are two crucial considerations. Hence, cautious selection of the matrix and filler is necessary when developing chitosan-based formulations. Enhancing mechanical and rheological characteristics is a prerequisite to improving the material's fabricability, making it easier to implement during wound dressing.

The voyage of chitosan-based formulations in tissue engineering and wound healing is far from over. As we look towards the future, numerous exhilarating directions and opportunities beckon researchers and clinicians alike. While chitosan formulations have displayed immense promise, advanced refinement in the design of the scaffold is necessary. Tailoring the scaffold's physicochemical characteristics, porosity, and framework to imitate the definite tissue microenvironment closely is a paramount objective. Developments in 3D printing and bioprinting technologies have the capability to generate highly complicated and patient-specific scaffolds.

Next, combining chitosan-based formulations with other advanced strategies is a fertile area for study. Synergistic strategy, for example, combining chitosan with stem cell therapy, may yield improvement in tissue regeneration and wound healing. Investigation into optimizing the interplay between chitosan scaffolds and diverse cell types could also guide improved tissue-specific results. Also, incorporating a range of bioactive molecules into chitosan-based preparation remains a propitious avenue. Growth factors, cytokines, and signaling molecules can be fine-tuned to develop a milieu that assists tissue regeneration or expedites wound healing. Exploiting the potential of nanoparticles, such as silver/zinc nanoparticles, for improved antimicrobial action is another area ripe for investigation. There is also a need to understand the complicated interplay between chitosan and the immune system. Controlling chitosan's immunomodulatory characteristics to fine-tune the immune response during tissue regeneration and wound healing could be a game-changer. Exploration in this area may guide to therapies that reduce scarring and inflammation. Though many investigations have shown favorable results *in vitro*, more investigations in animal models are pivotal. Transitioning chitosan-based formulations from the research lab to clinical practice is also a crucial step. Extensive clinical trials and strict regulatory processes are requisite to determine safety and effectiveness. Teamwork between academia, industry, and healthcare providers is essential to bridge the translational space.

Ultimately, the recent progress in chitosan-based formulations for tissue engineering and wound healing indicates a bright future for regenerative medicine. These novelties contain the assurance of revolutionizing patient care, furnishing solutions for difficult medical conditions, and enhancing the quality of life for innumerable humans. To comprehend this potential, continued association among researchers, practitioners, policymakers, and industry partners is paramount. By probing these future outlooks and driving the limits of knowledge, we can exploit the full capability of chitosan-based formulations to modify healthcare and healing in the years to come.

## Author contribution

Conceptualization and supervision: Sheersha Pramanik, A. Deepak, Stefano Belucci; resources: Sheersha Pramanik, Akanksha Aggarwal, Ammar Kadi, Kanchan Koul, Majid Alhomrani; literature review and writing—original draft preparation: Sheersha Pramanik, Akanksha Aggarwal, Ammar Kadi, Majid Alhomrani, Abdulhakeem S. Alamri; writing—review and editing: Sheersha Pramanik, Akanksha Aggarwal, Majid Alhomrani, Abdulhakeem S. Alamri, Kanchan Koul, Walaa F. Alsanie, A. Deepak, Stefano Belucci. All authors have read and agreed to the published version of the manuscript.

## Conflicts of interest

The authors declare no conflict of interest.
